# Scaling-up the use of sulfadoxine-pyrimethamine for the preventive treatment of malaria in pregnancy: results and lessons on scalability, costs and programme impact from three local government areas in Sokoto State, Nigeria

**DOI:** 10.1186/s12936-016-1578-x

**Published:** 2016-11-04

**Authors:** Nosa Orobaton, Anne M. Austin, Dele Abegunde, Mohammed Ibrahim, Zainab Mohammed, Jumare Abdul-Azeez, Hakeem Ganiyu, Zwalle Nanbol, Bolaji Fapohunda, Katherine Beal

**Affiliations:** 1 JSI Research & Training Institute Inc., Boston, USA; 2 JSI Malaria in Pregnancy Project (MiPP), Sokoto, Sokoto State Nigeria

**Keywords:** Malaria in pregnancy, IPTp-SP, Scale up, Integrated MNH, Primary health care, Human-centered design, Community engagement, Community-based health workers, Sokoto State, Nigeria

## Abstract

**Background:**

Intermittent preventive treatment of malaria in pregnancy with 3+ doses of sulfadoxine-pyrimethamine (IPTp-SP) reduces maternal mortality and stillbirths in malaria endemic areas. Between December 2014 and December 2015, a project to scale up IPTp-SP to all pregnant women was implemented in three local government areas (LGA) of Sokoto State, Nigeria. The intervention included community education and mobilization, household distribution of SP, and community health information systems that reminded mothers of upcoming SP doses. Health facility IPTp-SP distribution continued in three intervention (population 661,606) and one counterfactual (population 167,971) LGAs. During the project lifespan, 31,493 pregnant women were eligible for at least one dose of IPTp-SP.

**Methods:**

Community and facility data on IPTp-SP distribution were collected in all four LGAs. Data from a subset of 9427 pregnant women, who were followed through 42 days postpartum, were analysed to assess associations between SP dosages and newborn status. Nominal cost and expense data in 2015 Nigerian Naira were obtained from expenditure records on the distribution of SP.

**Results:**

Eighty-two percent (n = 25,841) of eligible women received one or more doses of IPTp-SP. The SP1 coverage was 95% in the intervention LGAs; 26% in the counterfactual. Measurable SP3+ coverage was 45% in the intervention and 0% in the counterfactual LGAs. The mean number of SP doses in the intervention LGAs was 2.1; 0.4 in the counterfactual. Increased doses of IPTp-SP were associated with linear increases in newborn head circumference and lower odds of stillbirth. Any antenatal care utilization predicted larger newborn head circumference and lower odds of stillbirth. The cost of delivering three doses of SP, inclusive of the cost of medicines, was US$0.93–$1.20.

**Conclusions:**

It is feasible, safe, and affordable to scale up the delivery of high impact IPTp-SP interventions in low resource malaria endemic settings, where few women access facility-based maternal health services.

*ClinicalTrials.gov Identifier* NCT02758353. Registered 29 April 2016, retrospectively registered

**Electronic supplementary material:**

The online version of this article (doi:10.1186/s12936-016-1578-x) contains supplementary material, which is available to authorized users.

## Background

In 2015, there were an estimated 214 million malaria cases and 438,000 malaria deaths in the world [1]. Sub-Saharan Africa accounted for 89% of global malaria cases and 91% of malarial deaths [2]. Nigeria alone accounted for almost 25% of malarial deaths in Africa [3]. In 2009, the Nigeria Federal Ministry of Health estimated that malaria was a direct contributor to 11% of overall maternal mortality, 25% of infant mortality and 30% of under-five mortality [3].

In Nigeria–with its estimated 6.35 million annual births in 2015—and in malaria-endemic settings, pregnant women, primipara in particular, are vulnerable to malarial infection [4–6]. On account of weakened immune systems, pregnant women are four times more likely than non-pregnant adults to suffer from symptomatic forms of malaria [6]. Furthermore, social factors such as unequal balance of power between women and men, constrain women’s equitable access to financing and health. This double burden undermines women’s ability to respond appropriately, and access prevention and treatment for malaria in pregnancy, even when services are available [7].

WHO-recommended strategies for the prevention and management of malaria during pregnancy comprise a three-pronged approach: (1) use of insecticide treated nets, (2) intermittent preventive treatment (IPTp), and (3) effective case management of malarial illness [8]. This approach was nuanced in 2012 with a call for countries to scale up IPTp [9]. WHO recommends the use of sulfadoxine-pyrimethamine (IPTp-SP), with a prescribed minimum of three doses during focused antenatal care visits. Each dose of IPTp-SP is expected to be recorded to enhance the monitoring of pregnant women that receive successive doses (i.e., IPTp-1, IPTp-2, IPTp-3, IPTp-4, etc.).

### Malaria and fetal growth

Malaria in pregnancy (MiP) has been shown to have an impact on fetal growth and birth outcomes [6, 10, 11]. Small size at birth has implications for newborn survival and long-term implications for a child’s growth and development [19]. Most studies on the impact of placental malaria on fetal growth have relied on birthweight as the outcome. In resource poor settings where many women give birth at home, mechanisms are scarce to weigh newborns [12–15], Sreeramareddy et al. have argued that in contexts where home births are prevalent, surrogate measurement approaches that do not require a weighing scale to determine low birth weight, are recommended for implementation [12].

### Malaria and stillbirths

There were an estimated 2.6 million (2.4–3.0 million) stillborn babies in the world in 2015 [16]. Ninety-eight percent of stillbirths occurred in low and middle income countries; Africa and Asia accounted for 77% of the global stillbirth burden [16]. With an estimated 40.1 stillbirths per 1000 live births, Nigeria has the second highest number of stillbirths in the world [17]. Nonetheless, the true magnitude of stillbirths in Nigeria is difficult to estimate in the absence of standardized national policies or protocols that enjoin facilities and communities to record, audit or to review causes of stillbirth [18].

Van Geertruyden et al. in a systematic review of 117 malarial studies conducted between 1948 and 2002 found that placental malaria was significantly associated with an increased odds of stillbirth (OR 2.19; 95% CI 1.49–3.22) [19]. However, Radeva-Petrova et al. noted that there were too few publications that studied a direct association between IPTp-SP and stillbirths [20]. The authors concluded that published studies were too “*underpowered to collect clinically important differences*” [20].

### IPTp-SP and ITNs for the prevention of malaria in pregnancy in Nigeria

Nigeria adopted the IPTp-SP strategy in 2005 [21]. SP was included in the national essential list of medicines as an over-the-counter medicine in 2005 [22]. Nigeria’s efforts to deliver IPTp-SP services during ANC care have not been successful or impactful [23]. In 2013, only 23% of women who had given birth in the two years preceding a survey received any dose of IPTp-SP when pregnant [24]. About 15% had received two doses of and just 6% received three doses of SP (SP3) [24]. In effect, the majority of pregnant women and their unborn babies in Nigeria are not adequately protected from placental malaria and its consequences [25].

A concern expressed in a 2013 WHO Consensus Statement on MiP, equally applicable to Nigeria, was that “*despite clear global gains in malaria control, the delivery of MiP interventions remains suboptimal in most endemic countries*” [9]. Too many women that attended antenatal care did not receive at least two doses of SP before delivery. There is now an urgent global call for new approaches and interventions to reduce missed opportunities and, in particular, to substantially increase the coverage of SP among pregnant women [9]. In 2013, the WHO Policy Brief for the implementation of IPTp called for research on “*innovative strategies to improve the delivery of IPTp*-*SP and malaria case management among pregnant women at the primary health centre level*”, and for “*innovative community strategies that do not detract from ANC services to increase IPTp coverage (such as community*-*based ANC outreach, promotion or distribution of IPTp)*” [26]. The policy brief also called for research into “*methods for using health system information systems for routine monitoring of IPTp*-*SP implementation and effectiveness*” [26].

A recent study in Southeastern Nigeria that utilized community-based workers to successfully distribute IPT-SP in community settings, reported that women in intervention areas were significantly more likely to ingest at least two doses of IPTp-SP [27]. However, the study did not test for the scalability of community-based distribution, nor did it track for associated newborn outcomes.

### The Sokoto State malaria in pregnancy project (MIPP)

MiPP was a yearlong project funded by Bill and Melinda Gates Foundation and implemented by JSI Research & Training Institute, Inc. that ended in December 2015. The project was undertaken in three local government areas (LGAs) of Sokoto state, Nigeria. The default IPTp-SP programme was a facility-only activity operated as part of its focused antenatal care services based on national guidelines [21]. The MiPP programme, to the best of our knowledge, is the first project in Nigeria and elsewhere, to include community-based delivery approaches and to track at scale, the number of IPTp-SP doses ingested by pregnant women, and associated newborn outcomes.

#### Sokoto State

Sokoto State is located in the North West Zone of Nigeria between longitude 11′′ 30–13′′ 50 and latitude 4′′–6′′. It borders Niger Republic to the north and Benin Republic to the northwest, Kebbi State to south and Zamfara State to the east. It has a land mass area of about 32,000 sq km, and consists of 23 local government areas and 244 political wards. Ward Development Committees (WDCs) are the smallest unit of governance which typically manage a revolving drug fund, supervise a cadre of community-based health volunteers (CBHVs), and oversee primary health facilities in their jurisdiction [28]. The population is predominantly rural, Muslim and consists almost entirely of Hausa/Fulani ethnic groups.

#### Sulfadoxine-pyrimethamine and insecticide-treated net use profile in Sokoto State

Malaria is seasonal in Sokoto State, with peak transmission from May to December. The highest point prevalence of parasitaemia—which mirrors rainfall patterns—is in August (59.5%), and the lowest is in March (9.18%) [29]. In 2013, the state prevalence rate of MiP was 9% and it ranged from 35% in the highest burden LGA to 1% in the lowest burden [30]. In 2013, 80% of women in the state did not use antenatal care in their last pregnancy—the largest non-use of ANC services by a state in Nigeria [24]. Of the 17.4% of women that obtained ANC from a skilled attendant, three in five received no SP whatsoever [24]. Less than 5% and less than 1% of all pregnant women in the state received SP2 and SP3 respectively in 2013 [24].

### Study overview

The study objectives were to:

1. Examine scale-up mechanisms that enable increased SP coverage through community-based primary health care delivery, without reducing facility uptake of SP.
2. Examine community acceptance of SP and the likelihood of long-term community-sustained demand.
3. Document associations, if any, between increased SP3 coverage and improved intrauterine conditions for newborn, as measured by head circumference increments and declines in still birth rates.
4. Estimate the costs of delivering SP at scale per woman for a three doses or higher regimen.

### The MiPP intervention

The intervention consisted of house-to-house distribution of SP to eligible pregnant women—administered through directly observed treatment (DOTs)—by trained community-based health volunteers (CBHVs). Community distribution of SP was limited to the first three doses of SP; SP4 and higher doses were intentionally designated for administration in a health facility, to promote the use of facilities. The MiPP intervention was twinned with an ongoing facility-based SP distribution as well as case management of suspected cases of malaria in health centers in intervention and counterfactual LGAs. Similarly, established and ongoing LLIN distribution continued in health facilities in both intervention and counterfactual LGAs.

#### Outcomes tracked

The programme tracked three principal outcomes. The first was the percentage IPTp-SP coverage among all pregnant women between April and November 2015 and by number of SP doses ingested. Head circumferences of live newborns was tracked, as a measure of intrauterine growth function. Head circumference was measured in centimetres, to the 10th of a centimetre, using a standardized protocol. A tape measure was placed above the ears and midway between eyebrows and the hairline to the occipital prominence at the back of the head. CBHV supervisors, all of whom were literate and numerate, carried out the measurements. Newborn head circumference, as a measure of intrauterine growth was tracked instead birthweight because it was considered less susceptible to the obesogenic effects of pregnancy [31]. Newborn head circumference has also been shown to be inversely related to and sensitive to malaria-associated lesions of the placenta [13, 31, 32]. Finally, in the context of Sokoto State, the measurement of head circumference was less intrusive and more readily managed by community health workers with adequate training.

Head circumference measurements were limited to live newborns whose mothers resided in one of four project LGAs, that were delivered—home or facility births—during the intervention period. Head circumference measurements were not undertaken in newborns whose mothers (a) recently migrated into the intervention area and had not been exposed to the intervention (b) older than seven days postpartum at the time of measurement, and (c) stillborn babies. Head circumference measurements were limited to those obtained within seven days postpartum to reduce temporal biases in our head circumference measurements. It was not culturally permissive to measure the head circumference of stillborn babies.

The third outcome was the incidence of stillbirths. Stillbirths were defined as deliveries that occurred after 7 months of gestation in which a baby was birthed without any signs of life (no breathing, no movement, and no sound) as reported by the mother or an informed family member.

#### Intervention LGAs and the counterfactual LGA selection

**Fig. 1:**
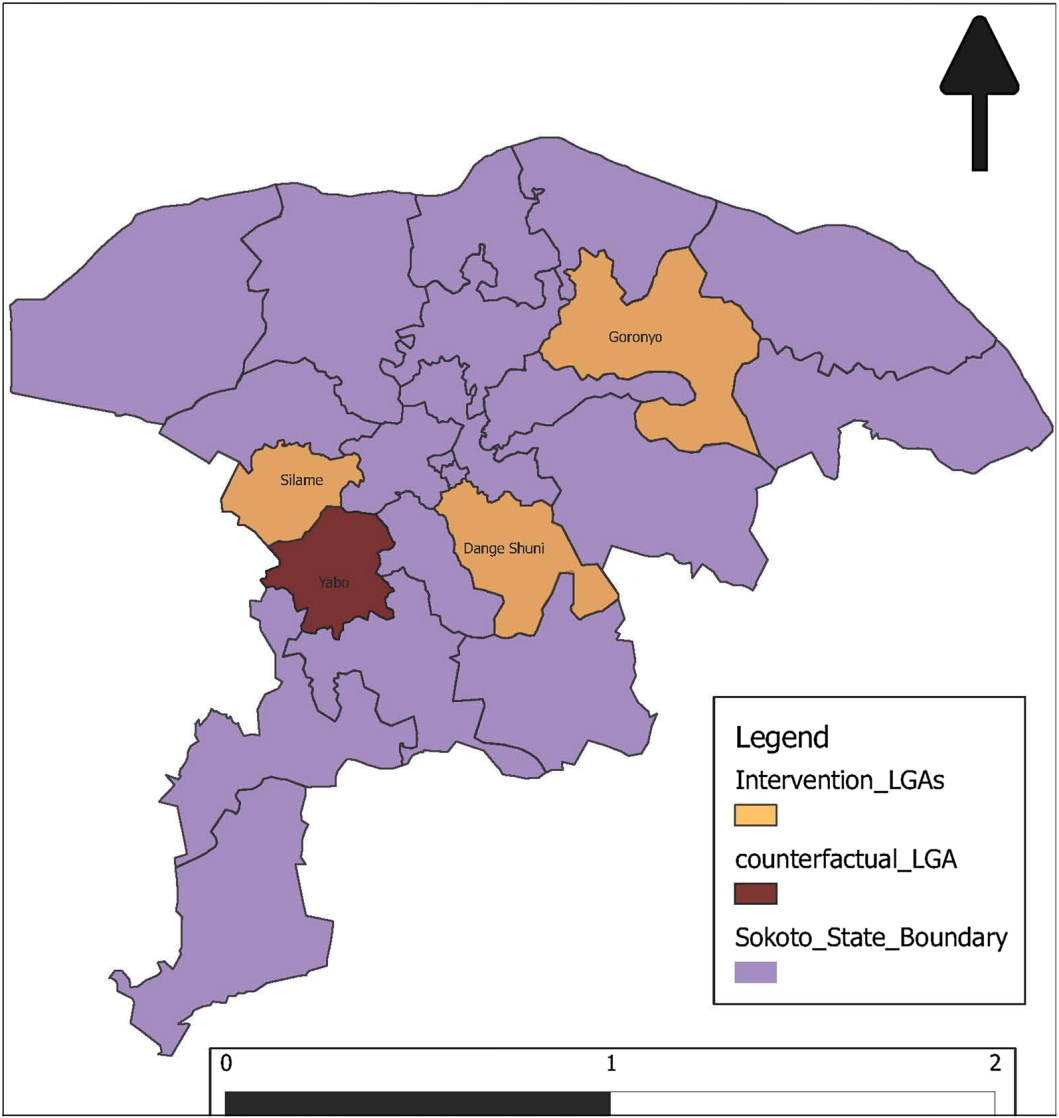
Map of intervention and counterfactual LGAs

Dange Shuni Goronyo and Silame (combined 2015 population, according to official Sokoto State estimates = 661,606) LGAs were purposively selected as intervention LGAs, and Yabo LGA, the fourth (2015 population, according to official Sokoto State estimates = 167,971), was selected as the counterfactual LGA. The selection criteria were that all LGAs had a high prevalence of malaria in pregnancy and that at one LGA each in the intervention group, was selected from each of the State’s three senatorial zones. Such political balance was considered important for the potential future adoption of study recommendations across Sokoto state. Figure 1 shows a mapping of the intervention and counterfactual LGAs.

#### Mapping households and identifying pregnant women in the community

In the three intervention LGAs, households were mapped and all women of reproductive age (WRA) were enumerated and registered in a household registration system (HRS) database. Designed with a household as the unit of analysis, the database contained compound numbers, household numbers, the number of WRA, pregnant women, and children under five. It also identified each woman—at the time of mapping exercise—that was pregnant, that was eligible for SP, when she got what SP dose, and where she resided. Any changes in these parameters in a given household were recorded into the Household Register Book (HRB), which was used to update the HRS on a monthly basis. Households were not mapped in the counterfactual LGA. Instead, official census projections were used to estimate the expected number of eligible pregnant women during the study period.

#### Implementing the malaria in pregnancy project

**Fig. 2:**
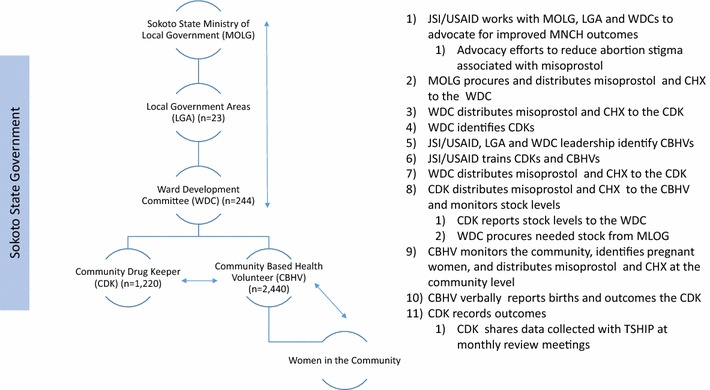
Sokoto State chlorhexidine–misoprostol distribution system

The IPTp-SP distribution built upon an existing community based distribution system introduced in 2013 by Sokoto State government with technical support from JSI/USAID. The community-based health volunteer system is comprised of 10 CBHVs per ward, which formed a cadre of 2440 CBHVs throughout the 244 wards in the State. CBHVs had already been trained to deliver the key counseling messages prescribed by the WHO and Nigeria’s National Primary Health Care Development Agency, and were assigned the additional task of delivering SP to women at home. CBHVs already had a system in place to implement an on-demand distribution of chlorhexidine gel 7.1% digluconate and misoprostol tablets to women that delivered their babies in home settings, which is shown in Fig. 2 [24, 33]. The study added five additional CBHV per ward to increase the geographic coverage of the intervention LGAs. With these additions, the number of CBHVs per 10,000 residents in the intervention LGAs of Silame, Dange Shuni and Goronyo were 10, 7 and 6, respectively. It was 7 per 10,000 in Yabo, the counterfactual LGA.

The medicine delivery component of the CBHV system had an inbuilt data collection system managed by a community drug keeper (CDK) and a supervising facility-based health worker to monitor distribution at the community level. Data captured in the outcome form included the condition of the newborn and mother at birth, of the newborn at birth—stillbirth or live birth—at days 7, 14 and 28 postpartum. The study team modified the outcome form to capture the number of SP doses a woman received in the intervention LGAs and data collection was managed by CBHV supervisors. In the counterfactual LGA, the outcome form was revised to exclude intervention-related questions that did not apply. It nonetheless inquired a pregnant woman’s primipara status, ANC status, SP doses taken, gestation at time of delivery, the state of newborns, and head circumference measurements. In the place of CBHV supervisors in the counterfactual LGA, 10 data collectors were recruited. These data collectors were titled as “home visitors” as they had no role in CBHV supervision. See Additional file 1 for examples of the modified outcome forms, for the intervention and counterfactual LGAs.

#### SP commodity logistics

In every ward in the intervention LGAs, one designated health facility served as a supply hub for SP intended for community distribution. The health facilities were selected based on their central proximity in a ward, possessing a good volume of clients, the availability of skilled service providers, and satisfactory medicines storage capacity. These health facilities were part of a long established statewide supply grid for malaria commodities including artemisinin-based combination therapy (ACT) medicines, rapid diagnostic testing kits (RDT), (LLIN) and SP. These commodities were provided by US Presidential Malaria Initiative (PMI) through the USAID/JSI DELIVER Project. Health facilities in the counterfactual LGAs were similarly supplied with identical malaria commodities. Two service providers from each designated health facility were co-trained with CBHV supervisors on the community distribution process and on the Nigeria National Malaria policy guidelines—on SP dosing, malaria case management—referrals and documentation of services rendered into registers and commodity stock books. Health facilities supplied SP tablets to CHBV supervisors who in turn distributed them to the CBHVs in the wards. Within each ward, a CBHV regularly covered a defined catchment of compounds/households.

#### SP distribution process

The scale-up of community distribution of SP was trialed in three wards (one ward per LGA) for 1 week, and reached all known pregnant women eligible for SP (n = 264). During the trial, it was learned that the system used to identify households caused avoidable delays in tracking women. It did not work for the CBHVs, most of whom were unable to read or write. It was successfully replaced at scale up by the use of simple pictograms that uniquely identified households. Additional file 2 provides a sample of the pictograms used by the CBHV’s. CBHVs and their supervisors met weekly, and updated their lists of eligible pregnant women for IPTp–SP, and made quantifications of SP doses needed.

**Fig. 3:**
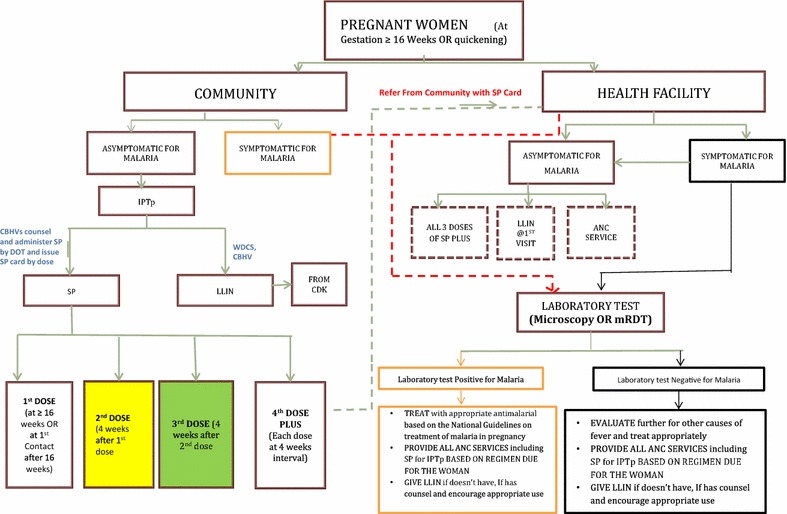
Decision tree for SP dosage and referral

At the household level, CBHVs counselled pregnant women and inquired about any symptoms of malaria, the gestational age of pregnancy and presence of quickening, history of SP administration within last 4 weeks, a history of reaction to sulphur-containing drugs. CBHVs counseled women on the benefits of IPTp-SP, of LLIN utilization, good nutrition and the importance of ANC visits. Eligible and healthy pregnant women were given a dose of SP administered via DOTs and told the date of their next monthly dose. CBHVs issued a colour-coded card linked to a specific dosage administered—white for 1st dose, yellow for 2nd dose and green for the 3rd dose. Each SP card was pre-recorded with a given woman’s compound number, household number and her unique number. Mothers were advised to bring their SP cards to ANC clinic visits. This setup enabled more reliable data exchange with health workers in health facilities on what doses a woman had received at home. Examples of the cards used to track SP doses can be found in Additional file 3. Figure 3 shows the decision tree for IPTp-SP dosage and referral protocol applied in the MiPP project.

#### Review meetings

Two sets of review meetings—a monthly one with CBHVs and their supervisors and a bi-monthly one with LGA officers—were regularly conducted. At monthly ward meetings, CBHVs with their supervisors, service providers and WDC members, LGA RBM officer, LGA M&E officer and project team members reviewed the month’s work and exchanged experiences. Three wards were clustered per meeting for cross-learning. One outcome of such meetings, at the behest of community leaders, was the introduction of outreach ANC services to underserved hard-to-reach areas. ANC clinics run by community health extension workers and midwives were held at least thrice there so that three doses of SP were delivered.

**Fig. 4:**
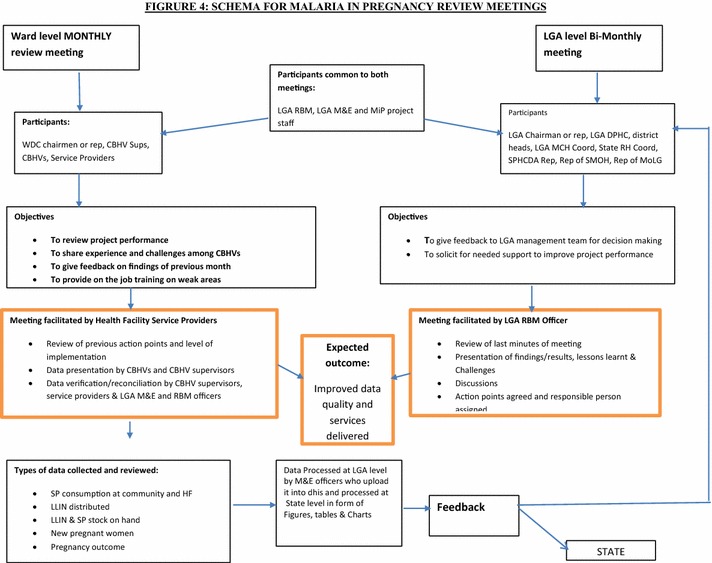
Planning schema for MiPP project review meetings

At LGA bi-monthly review meetings, feedback was provided to LGA level stakeholders, and lessons for future use beyond the project, were drawn. Participants at these meetings included the LGA chairman or his designate, the LGA primary health care director and team (the MCH coordinator, RBM officer and M&E officer), district heads from the LGA, WDC representatives, representatives of the State Primary Health Care Development Authority, representatives of the Ministry of Local Government, representatives of facility-based service providers, three representative CBHV supervisors and the project staff. Figure 4 shows the planning schema and participants for the MiPP review meetings.

#### Monitoring the safety of IPTp-SP delivery

During the project, all service providers searched proactively for reports of adverse reactions to SP use in pregnant women. All CBHVs and their supervisors were trained on how to ask about any adverse reactions and how to document them if they occured. The imperative that there be a clear cut history of quickening as a prerequisite for SP use in pregnant women, was reinforced in trainings and throughout all review meetings and supervision encounters at ward, facility and LGA levels. WDC members were also asked to report any suspected adverse reactions to SP in their communities. At the health facility where such practices were the norm, service providers regularly checked for suspected reactions for onward referral to a secondary referral health facility. As found in other studies, IPTp-SP was well tolerated; there were no reported cases of severe adverse reaction to SP [34].

#### Ensuring data quality on SP consumption and reporting at facility and community levels

Data quality checks of SP doses administered were performed on health facilities’ and CBHV Supervisors’ records. At the facility level, daily SP services are typically summarized into a monthly summary form (MSF), which is entered into a cloud-based national DHIS2 database that constitutes Nigeria’s national monthly summary health indicators reporting system. The MSF was used as the standard with which to measure accuracy. Within the DHIS2, each facility that reports data is named and so it is possible to compare a facility’s DHIS2 data with what was reported in the same facility’s MSF. Between June and November, monthly reviews of the DHIS2 reported data in a given facility was cross-checked with the MSF. Inconsistencies were compiled and shared with a team of the LGA Monitoring and Evaluation Officer (LGA M&E Officer), the LGA Roll Back Malaria Officer (LGA RBM Officer) and the team leader of health facilities, for resolution. For example, the problem of non-reporting by some facilities was traced to insufficient coordination between the LGA RBM Officer and M&E Officers and to lack of transportation to reach out to distally located facilities. Health facilities in the intervention LGAs added up SP doses served in their catchment communities to the facility data.

In the intervention LGAs, the HRB master list, an aggregation of individual household HRB, contained the most up-to-date information of WRA, pregnant women and SP doses given. HRB was the source of information for CBHVs and their supervisors to prepare and regularly update lists of eligible women. CBHVs and their supervisors documented their list of women reached with SP in the HRB with which dose and when. CBHV Supervisors prepared monthly summary forms from these activities. The HRB master list was compared each month with the CBHV Supervisors’ monthly summary form. Discrepancies were addressed by CBHV supervisors at follow up home visits.

#### Tracking birth reporting, birth outcomes and head circumference measurements

Twelve teams of four data quality auditors, independent of other project staff, were recruited to track data quality. Each team comprised of three females and one supervisor. Over the life of the project, the teams visited all the women recorded with at least one birth—that occurred during the project—in the 42 wards of the three intervention and one counterfactual LGAs. Information on omitted mothers and births was sought for and collected. Mothers—or an informed family member in the event of a maternal death—if a CBHV and CBHV Supervisor visited, the status of newborns, alive or stillborn, and if head circumference was measured within seven days among live births. With mothers’ responses as the gold standard, births, status of births, and confirmed HC measurements were verified. Overall, a 3.7% error rate was found with facility-based SP records, 2.5% error rate with community-based SP records. There was a 17% underreporting of live births and 2.1% error rate with the correct recording of births and outcomes. These were all addressed prior to data analysis.

## Methods

### Data and methodology for the coverage and scale of SP doses

From the HRS database, 25,572 women who were eligible for SP between April and November in the three intervention LGAs, were identified. Based on actual numbers, 94% of pregnant women in the intervention LGAs became eligible for SP1 between April and November. In the intervention LGAs, the number of pregnant women eligible to receive SP1, was used as a denominator to calculate SP1, SP2 and SP3+ coverage (Table 4).

In the counterfactual group, the number of pregnant women eligible to receive SP1 over the life of the project was estimated. Using the official population estimate for Yabo LGA, from the Sokoto State Government (167,971) and assuming that 5% of the population was pregnant, there were an estimated 8399 pregnant women in Yabo, in 2015. Prorating that number for the 8 months of the project, there were an estimated 6299 pregnant women in Yabo between April and November 2015. Assuming Yabo would have the same percentage of women eligible for SP1, as found in the intervention areas (94%) it was estimated that there was a total of 5921 women eligible for SP1 during the project lifespan (Table 4). This estimate as the denominator to calculate SP1 and SP2 coverage.

Univariate and bivariate analyses were performed to compare intervention and counterfactual LGAs on the number of SP doses and source of SP. Analyses were conducted in Excel spreadsheet^®^.

### Newborn head circumference data and methodology

Programmatic data extracted from outcome forms were available for 9241 live births in intervention and counterfactual LGAs between April and November. Head circumference (analyzed in mm) of live newborns measured within 7 days of birth, was available for 6720 (73%) of live births. Head circumference data were missing for 2521 live newborns. Of these, 1721 (19%) mother-newborn dyads missed initially, were identified during the data quality review in October. For the remaining 800 newborns, head circumferences were not measured within 7 days postpartum.

Independent variables used in this analysis were guided by prevailing epidemiological evidence base and by what was feasible to collect by CBHV supervisors. These included the sex of newborn, gravidity of mothers, successive doses of SP, exposure to ANC, month of delivery and gestational age at birth. Globally, female newborns have smaller head circumferences than male newborns [15]. Primigravida women are at higher risk for placental malarial infection, [35]. Table 1 presents how each of these variables was coded in the analyses.

**Table 1:** Variables used to understand the impact of SP interventions on head circumference among 6720 live newborns, born between May and November 2015

Variable name	Definition	
Head_circum	Head circumference, at birth, in mm	n = 6720 Continuous
Girl	1 = Female	n = 6712
	0 = Male	
SPFREQ	0,1,2, 3 + doses of SP	n = 6720
Intervention	1 = DANGE SHUNI, Goronyo or Silame LGA, where community based SP distribution occurred	n = 6720
	0 = Yabo, where no community based SP distribution occurred	
Primi	1 = Primigravida	n = 6711
	0 = Multigravida	
Month of birth	April–November 2015	In the unadjusted analyses, all earlier months are compared to November. In the adjusted analyses, this variable was used as a continuous variable n = 6720
Gestational age at birth (months)	2 = 10	n = 6719
	1 = 9	
	0 = 8	

Univariate analyses tested for any associations between a given independent variable and the mean head circumference of live newborns. Unadjusted t tests were used to assess any differences within each predictor variable and newborn head circumference. Unadjusted tests of correlations between mean head circumference and doses of IPTp-SP, and month of birth, were performed. Mean newborn head circumferences were used to assess correlations in the number of IPTp-SP doses over the project period in the intervention and counterfactual LGAs. A multivariate linear regression model was used to test for the impact of SP doses and other variables on head circumference. Analyses were performed with Excel^®^ and SAS v. 9.4.

### Stillbirth data and methodology

Data extracted from outcome forms were available for 9453 term births in both the intervention and control LGAs between April and November. To examine the impact of IPTp-SP doses on the incidence of stillbirths, all confirmed pregnancies that ended in miscarriage and abortion, or were delivered before 8 months of gestational age (n = 99) were excluded. If the newborn was stillborn, it was coded as “1”; if it was a live birth, it was coded as “0.”

Stillbirth rates (SBR), per 1000 births, and correlations between SBR and doses of SP were calculated. Unadjusted and adjusted logistic regression modelling was used to predict the odds with 95% confidence intervals, of having a stillbirth among women who ingested different doses of SP, according to exposure to intervention, those who attended at least one ANC visit, gravidity of mothers, those who gave birth in a facility vs those who gave birth at home, and those who gave birth later (July–November) vs. those who gave birth earlier (April–June). Multivariate logistic regression analysis was used to assess whether these associations would hold after controlling for other variables in the model. Analyses were conducted in Excel^®^ and SAS v9.4. Table 2 presents each variable as coded in the analyses.

**Table 2:** Variables used to understand the associations between SP interventions and stillbirths between April and November 2015 (n = 9453)

Variable Name	Definition	N and notes
Stillbirth	1 = Stillbirth	A stillbirth was defined as an infant born at least 8 months or more of estimated gestational age, who showed no signs of life at birth
	0 = Live birth	Births that occurred before 8 months gestation were coded as missing (miscarriage/abortion)
SPFREQ	0,1,2, 3+ doses of SP	
Intervention	1 = DANGE SHUNI, Goronyo or Silame LGA, where community based SP distribution occurred	
	0 = Yabo	
Any ANC	1 = Yes	
	0 = No	
Primi	1 = Primigravida	n = 9438
	2 = Multi	
Place of delivery	1 = Health facility (hospital, health center/post)	
	0 = Home	
Month of birth	1 = July–November	Month of birth
	0 = May, June	

### Costing data and methodology

Nominal cost and expense data in 2015 Nigerian Naira (NGN) directly related to community and facility distribution of SP in the intervention and counterfactual LGAs were obtained from project records and other sources. The cost estimates obtained are what it would cost the state government and LGAs as de jure providers of primary health care in Nigeria, to deliver SP-related services at both the community and facility level, including start-up costs. Estimates were limited to a 12-month horizon.

Different degrees of contributions by each service component at facility and community levels, towards the delivery of SP at facility and community levels, were assumed. Table 3 lists the assumptions about the magnitude of contributions by each level of care to SP distribution. For this purpose, six cost centers were included in the analysis: health facility, LGA technical administration, CBHV supervisors, WDC, CBHV, and logistics for SP distribution. Table 3 provides a summary of each cost center, and their relative contribution towards SP distribution the activities involved; these were costed. Twenty-two work days per month were assumed. Published government salary schedules were used to compute government officers’ salary costs. Governments officers’ salary costs included time spent at monthly LGA level review meetings in each LGA attended by representatives of wards. At the community level, costs were attributed to WDCs and to CBHVs. There was a WDC and one CBHV supervisor in each ward. WDCs supported CBHVs in the distribution of SP and supervised CBHV, as well as ad hoc community meetings that were called to tackle issues that could undermine the demand or supply sides of the programme. CBHV-related costs also included level of effort, transportation for SP distribution and monthly rentals of meeting rooms for ward-level CBHV review meetings also attended by LGA officials. Costs associated with the transportation and distribution of SP to 42 health facilities were captured in central storage costs.

**Table 3:** Cost centers by level of care and magnitude of their component costs associated with SP distribution

Cost center	Level	Description	Components costed (magnitude)
Health facility	Health Facility	SP distributed through ANC. Hub for community distribution and receipt of community consumption data	Rent and utilities (10%) M&E documentation tools (10%) Two health staff that operate ANC (50%) In-facility storage (10%)
Local Government Area Council	LGA-wide, all levels	Supervision and program oversight including quality assurance, data management and coordination	Meeting venue (100%) LOE for nine key personnel at all levels who contribute supervisory and oversight roles and collaborate in the program (5% time)
CBHV Supervisor	Community/ Facility	Technical supervision of CBHV, assuring SP availability to CBHV and data-driven accountability to WDC and HF	LOE (50%) Transport costs of SP from HF-CBHV (100%) Monitoring and Supervision (100%)
CBHV	Community	Oversight and coordination of community-level operations	CBHV LOE (50%) Transport cost for SP distribution (100%) Transport costs for home visits (100%)
Ward Development Committees	Community	Community mobilization and sustaining program momentum	Meeting attendance costs for WDC member (30%) Meeting attendance cost of CBHV supervisor of ward (30%) Supervision and monitoring of CBHV (100%) Meeting/dialogue (100%) Meeting venue (100%)

Two ratios were calculated: cost per dose and cost per woman served, disaggregated by number of SP doses in the intervention and counterfactual group. Ratios were obtained from annualized costs derived in each LGA, intervention and counterfactual, the total as well as the disaggregated number of SP doses distributed, and from the total number of women served.

## Results

### Findings related to coverage and scale-up of SP use

A combined total of 60,428 compounds which comprised 92,240 households with a combined population of 524,580 residents were mapped and enumerated in the three intervention LGAs and registered into the database. According to Sokoto State official figures, there was an estimated population of 167,971 in the counterfactual LGA (Table 4). A total of 114,842 women of reproductive age were registered in the intervention LGAs. Throughout the intervention period, April-November, a total of 25,572 pregnant women in the intervention LGAs and an estimated 5921 in the counterfactual LGA were eligible to receive SP1 (Table 5).

**Table 4:** Determining the number of women eligible for SP1 between April and November 2015, and differences between project mapping and official projections

LGA	Official projected population in 2015	Percentage pregnant women per annum (assuming 0.05 of Pop)	Estimated number of pregnant women over 8 month period	Enumerated pregnant women from the MiPP database	% Difference between MiPP database and projected population of pregnant women	Women that became eligible for SP1 Between April and November 2015	% Eligible for SP1 between April and November in MiPP database (%)
Dange Shuni	255,908	12,795	9597	9550	0%	9136	96
Goronyo	257,984	12,899	9674	11,231	14%	10,850	97
Silame	147,715	7386	5539	6251	11%	5586	89
Yabo	167,971	8399	6299	NA	NA	5921^b^	94^a^
Total	829,577	41,479	31,109	27,032	8%	31,493	94

^a^Mean percentage of women becoming eligible for SP1 in the intervention LGA’s

^b^Estimated using the mean percentage of pregnant women that became eligible for SP1 in the intervention LGAs

**Table 5:** Number and percentage of eligible women who received SP1, SP2 and SP3 by LGA

	Dange Shuni	Goronyo	Silame	Intervention combined	Yabo	Total
Pregnant women eligible for SP between April and November 2015	9136	10,850	5586	25,572	5921	31,493
Number of SPI doses consumed	7408	10,893	6013	24,314	1527	25,841
% Given SP1	81	100	108	95	26	82
Number of SP2 doses consumed	5334	7304	4656	17,294	767	18,061
% Given SP2	58	67	83	68	13	57
Number of SP3+ doses consumed	2922	5381	3128	11,431	0	11,431
% Given SP3+	32	50	56	45	0	36
Total number of doses SP doses given	15,664	23,578	13,797	53,039	2294	55,333
Mean doses of SP delivered to eligible women	1.71	2.17	2.47	2.07	0.39	1.76

**Fig. 5:**
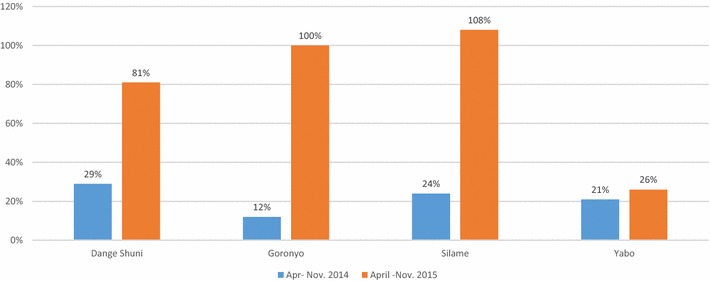
Percentage coverage of pregnant women who took SP1 in three intervention LGAs and one counterfactual LGA, Sokoto State, Apr–Nov 2014 and Apr–Nov 2015

Table 5 shows that between April and November 2015, the percent of eligible women that got SP1 averaged 95% in the intervention LGAs—ranging from 81% in Dange Shuni to 100 and 108% respectively in Goronyo and Silame—compared to 26% in the counterfactual (Figure 5). The high SP1 coverage in Goronyo LGA was also influenced by a test conditional cash transfer programme to encourage women to attend ANC clinics—including those from neighboring LGAs—that was also in effect during the study. In Silame LGA, there were a number of households whose pregnant women were served with SP and not captured in the HRB due to delays in enumeration. Consequently, these were not included in the denominator, and quite likely yielded an overestimated SP1 coverage.

Table 5 shows that the SP2 coverage in the intervention LGAs was 68%—and it ranged from 58% in Dange Shuni to 83% in Silame LGAs–compared to 13% in the counterfactual LGA. The SP3 coverage in the intervention group was 45%–it ranged from 32% in Dange Shuni to 56% in Silame—compared to zero percent in the counterfactual. Data on SP3 was unavailable for the counterfactual as the DHIS2 does not collect data on SP3 doses and higher.

Figure 5 compares SP1 coverage in intervention and counterfactual LGAs in 2014 and 2015. The percent of women that received SP1 in the intervention did not differ from those that did in the counterfactual in the 12 months that preceded the 8 month (April–November) study period, and it averaged 22%.

**Fig. 6:**
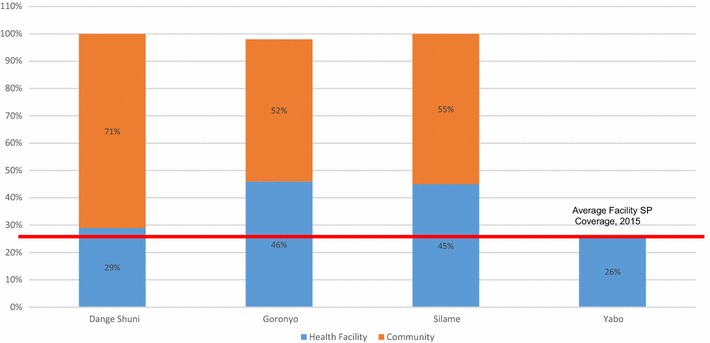
Percentage coverage of pregnant women who took SP1 in three intervention LGAs and one counterfactual LGA by source April–November 2015

Figure 6 shows that across the three intervention LGAs, between 55 and 71% of all SP1 through SP6 doses consumed were obtained from community channels. A 26 percentile line drawn through the bar graphs of the intervention LGAs indicate that relative to the 26% coverage in the counterfactual LGA which was an all-facility distribution scheme, there was a proportional increase in health facilities as a source of SP consumed in intervention LGAs. The introduction of community SP distribution may have helped increase SP uptake in facilities in at least two intervention LGAs.

**Fig. 7:**
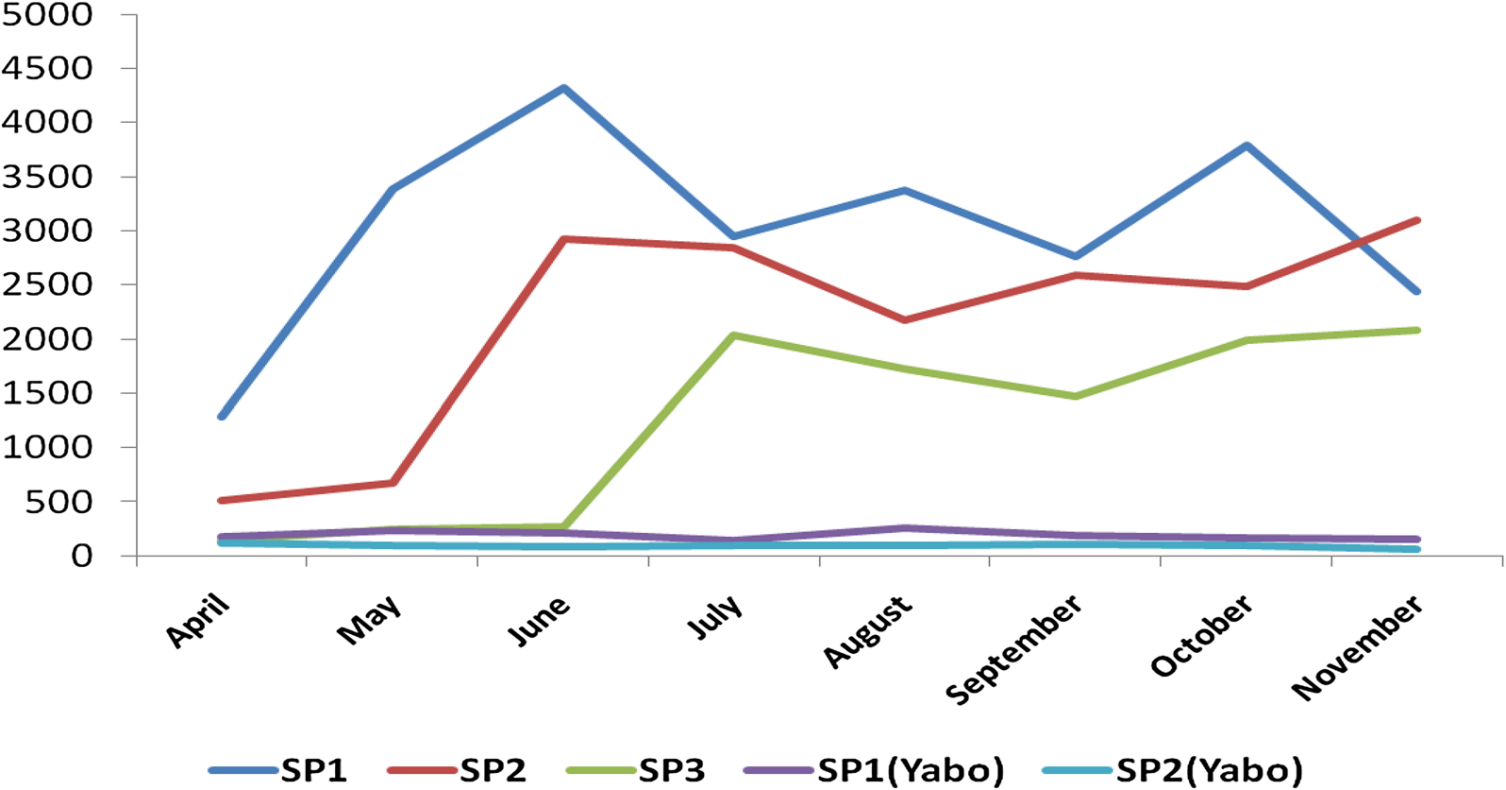
Monthly number of pregnant women who took SP1, SP2, SP3 in three intervention LGAs and one counterfactual LGA, Sokoto State, April–November 2015

The monthly number of women that consumed SP1, SP2 and SP3 in the intervention LGAs, and women that consumed SP1 and SP2 in the counterfactual LGA were plotted (see Fig. 7). There was an early sharp increase in SP1 users suggestive of rapid community adoption aided an already well-established community distribution network. There was a subsequent marked increase in the uptake of SP2 and SP3 suggestive of sustained continuity of use. From June onwards, there was a pattern of convergence between SP1 and SP2 consumption, and by November, the number of women that got SP2 exceeded those that got SP1—another likely indication of successful follow up of pregnant women that held over time. It also reflects a persisting capability of women to demand SP3, and the supply system to meet such demand. In Yabo LGA, the counterfactual, the trend lines of the numbers of women that received SP1 and SP2 per month were much lower and changed little throughout the study period.

### Findings related to newborn head circumference

**Fig. 8:**
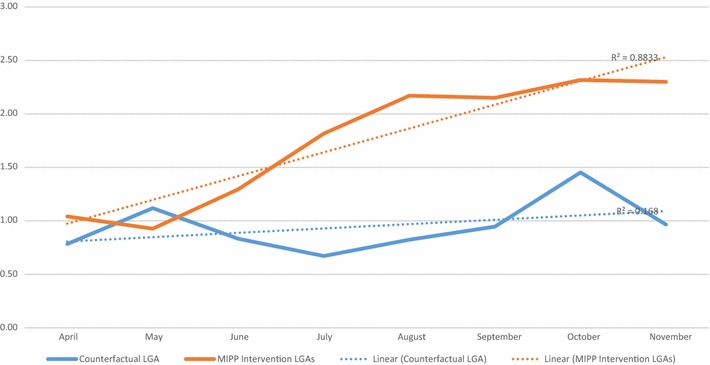
Mean doses of SP between April and November 2015 in the MiPP intervention and counterfactual LGAs

**Fig. 9:**
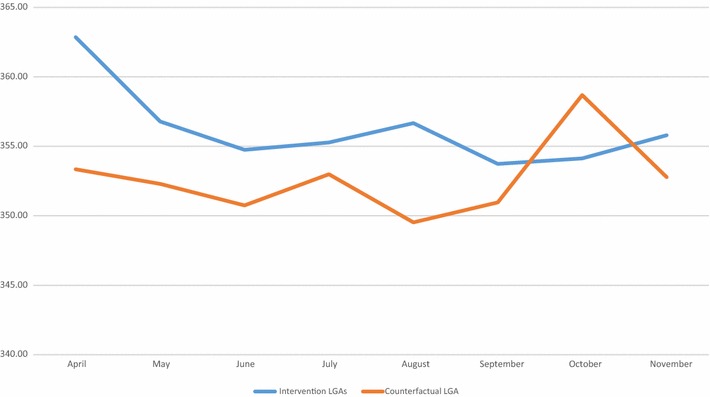
Mean head circumference in the intervention and counterfactual LGAs by birth month (April–November 2015)

Data on head circumference were significantly more likely to be missing in the intervention (missing n = 2512) versus the counterfactual LGAs (missing n = 9) (Chi-sq p < 0.0001). These were babies who were past 7 days postpartum whose head circumferences were not measured, and therefore excluded. There was a strong correlation between later birth months and increasing number of IPTp-SP doses (r^2^ = 0.88) in the intervention LGAs. This indicates that more women proactively enrolled earlier in their pregnancies when the community-based distribution of IPTp-SP commenced on April 28, 2015. They were also more likely to have received all three recommended doses of IPTp-SP, than those who enrolled when their pregnancy was in far advanced stages of gestation. This correlation was weak in the counterfactual LGA where there no community mobilization effort (r^2^ = 0.17) (Fig. 8). The mean head circumference among all live newborns was 355 mm (see Table 6). Table 6 shows that babies born later in the year had smaller head circumferences than those born in April. A month on month decline in mean head circumference was observed in both the intervention and counterfactual LGAs between April and November. In all but 1 month (October) head circumferences were consistently larger in the intervention LGAs (Table 7) (Fig. 9).

**Table 6:** Mean head circumference by month of birth, between April and November 2015

Month of birth	n	Mean	SD
April	755	360.50	22.89
May	887	355.49	20.92
June	838	353.06	20.86
July	766	354.53	23.78
August	809	354.31	22.61
September	860	352.98	26.17
October	959	355.63	20.88
November	846	354.83	21.14
Total	6720	355.11	22.52

**Table 7:** Mean head circumference by month of birth between April and November 2015 in the Intervention and counterfactual LGAs

Month of birth	Intervention LGAs			Counterfactual LGA			Pr > |t|
	n	Mean	SD	n	Mean	SD	
April	567	362.86	23.94	188	353.35	17.61	<0.0001
May	631	356.79	22.20	256	352.30	16.99	0.00
June	485	354.75	22.85	353	350.75	17.52	0.01
July	515	355.28	25.63	251	352.99	19.41	0.21
August	542	356.67	23.42	267	349.53	20.06	<0.0001
September	626	353.73	27.89	234	350.97	20.81	0.17
October	643	354.13	22.04	316	358.69	17.95	0.00
November	574	355.80	21.24	272	352.78	20.80	0.05

**Fig. 10:**
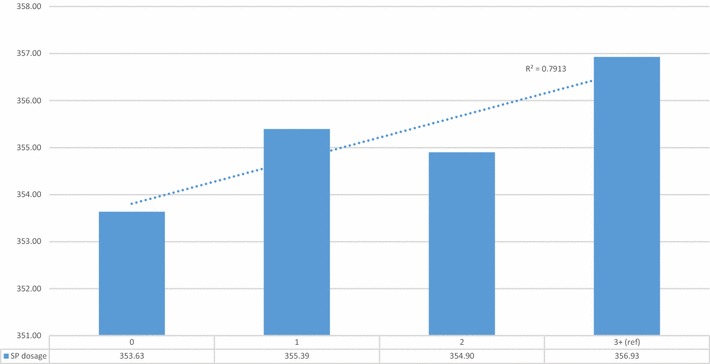
Mean newborn head circumference in mm by number of SP doses during pregnancy, for births between April and November 2015

Table 8 presents findings from the univariate analyses. As expected, female newborns had significantly smaller head circumferences than male newborns. There was a linear increase in mean head circumference with each additional SP dosage (r-sq. = 0.79) (Fig. 10). Larger and statistically significant mean head circumferences were observed among newborns whose mothers had at least one ANC visit, relative to those whose mothers’ had none. There was no significant association between being primigravid and a smaller head circumference. Newborns in the intervention LGAs had significantly larger head circumferences compared to those in the counterfactual LGA.

**Table 8:** Unadjusted mean head circumference (HC) in millimeters (mm) among live newborns born between April and November 2015, and differences in mean HC by available variables

	n	Mean HC in mm	SD	Pr > |t|
Sex of infant				
Female	3235	353.90	22.17	<0.001
Male (ref)	3477	356.30	22.76	
Intervention				
Yes	4583	356.20	23.88	<0.001
No (ref)	2137	352.70	19.07	
SP dosage				
0	2258	353.63	23.51	<0.0001
1	871	355.39	23.14	0.10
2	1682	354.90	21.37	0.01
3+ (ref)	1909	356.93	21.89	
Primigravida				
Yes	1225	354.30	22.57	0.18
No (ref)	5486	355.30	22.51	
At least 1 ANC visit				
Yes	3805	356.00	20.19	0.00
No (ref)	2915	354.00	25.20	
Month of birth				
April	755	360.50	22.89	<0.0001
May	887	355.49	20.92	0.54
June	838	353.06	20.86	0.11
July	766	354.53	23.78	0.79
August	809	354.31	22.61	0.64
September	860	352.98	26.17	0.09
October	959	355.63	20.88	0.45
November (ref)	846	354.83	21.14	
Gestational age at delivery (months)				
8	18	342.50	26.44	0.00
9	6588	355.06	22.57	0.01
10 (ref)	113	360.26	17.29

Table 9 presents the adjusted associations between each of the predictor variables and live newborn head circumference. On average, female newborns had head circumferences that were 2.33 mm smaller than male newborns. Newborns whose mothers had three or more doses of IPTp-SP had a significantly larger head circumference than newborns whose mothers ingested 0, 1 or 2 doses. On average, the newborns of mothers who had attended one or more ANC visits had a significantly larger head circumference, of 2 mm, than babies whose mothers had no ANC visits. Women who resided in the intervention LGAs had newborns with significantly larger mean head circumference at 2.88 mm than those living in the counterfactual LGA. There was a significant, negative, linear trend in head circumference as birth months progressed by 0.64 mm/month from April to November. There was no significant association between mothers being primigravida and a lower newborn head circumference. Newborns that were delivered at 8 months of gestation had on average a head circumference 17.83 mm smaller than those who delivered at 10 months of gestation.

**Table 9:** Adjusted associations between selected variables and the mean head circumference (HC) of newborns, between April and November 2015

	Estimate	Standard error	Pr > |t|
Intercept	363.78	2.77	<0.0001
Sex of infant			
Female	−2.33	0.55	<0.0001
Male (ref)			
Intervention			
Yes	2.88	0.66	<0.0001
No (ref)			
SP dosage			
0	−2.87	0.87	0.00
1	−1.88	0.93	0.04
2	−2.55	0.78	0.00
3+ (ref)			
Primigravida			
Yes	−1.16	0.71	0.10
No (ref)			
At least 1 ANC visit			
Yes	2.01	0.62	0.00
No (ref)			
Month of birth	−0.64	0.13	<0.0001
Gestational age at delivery (months)			
8	−17.83	5.68	0.00
9	−3.73	2.14	0.08
10 (ref)			

n = 6702; F test p < 0.0001; Adj. r-square 0.017; RMSE: 22.6

To test for collinearity between ANC attendance, gestational age and SP dosages two interaction terms were constructed. These interaction terms were used to test for any distinct impact of ANC and gestational age at SP0, SP1, SP2 (relative to SP3) on newborn head circumference. None were significant or of any added explanatory value. This suggests that the effects of SP dosage, gestational age, and ANC attendance on changes in newborn head circumference appeared to operate independently of each other.

The mean head circumference by month of birth in the counterfactual LGA, showed a surge in October in both the number of SP doses and head circumferences. This coincided with data quality audit visits, that likely increased community awareness of SP. October data were excluded and the unadjusted and adjusted analyses were re-run. All estimates increased in magnitude, and with stronger statistical significance. There was also a stronger SP linear association between higher SP doses and increased head circumference, as well as a stronger intervention effect. Therefore, there may have been a “community mobilization” effect of data quality audits carried out in the counterfactual group. See Additional files 4 and 5.

### Findings relating to stillbirths

**Fig. 11:**
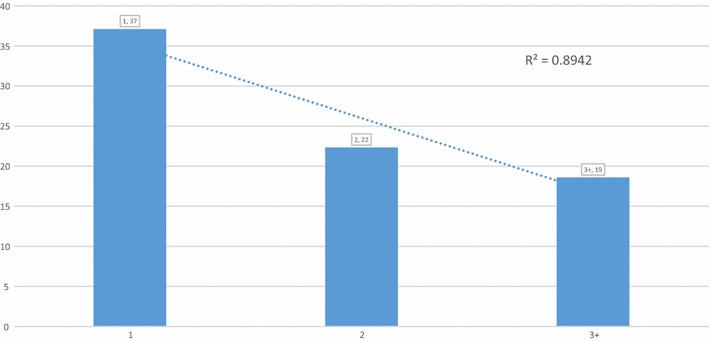
Stillbirth rate by 1, 2 and 3+ doses of SP, for births between April and November 2015

There were 213 stillbirths out of 9453 births that occurred after 7 months of gestation, equivalent to an overall stillbirth rate (SBR) of 23 per 1000 births. The SBR by dose of SP was calculated, and unexpectedly showed that women who ingested zero doses (SBR = 21 per 1000 term births) had a lower SBR than those who had ingested SP1 (SBR = 37 per 1000 term births) or SP2 (SBR = 22 per 1000 term births). (Table 10) Among women who ingested at least one dose of SP, a strong correlation between increasing SP doses and declines in SBR was observed. (r-sq = 0.90) (Fig. 11).

**Table 10:** Frequency of Stillbirths by number of SP doses, and Stillbirth rate (SBR) between April and November 2015

Status at birth	Number of SP doses				
	0	1	2	3+	Total
Livebirth	2644	1142	2233	3221	9240
Stillbirth	57	44	51	61	213
Total births	2701	1186	2284	3282	9453
SBR	21	37	22	19	23

A stillbirth was defined as an infant born at 8 months or more gestational age, who showed no signs of life at birth

*SBR* the number of stillbirths per thousand births

Table 11 presents the unadjusted odds of women having a stillbirth between April and November. The odds of having a stillbirth were higher among women who ingested less than three doses of SP, but was only statistically significant when contrasted to SP1 (OR 2.04). Being resident in the intervention LGA versus the counterfactual LGA was not a significant predictor of a woman having a stillbirth. Newborns that were delivered later in the study were no different from those born earlier in the odds of being a stillbirth. Any ANC attendance significantly reduced the odds of a woman having a stillbirth (OR 0.57). Primigravid women (OR 1.87) and those mothers who gave birth in a facility (OR 1.86) had significantly higher odds of a stillbirth compared to multigravida women and those who gave birth at home.

**Table 11:** Unadjusted odds of having a stillbirth (vs. a live birth) between April and November 2015 in four LGA’s in Sokoto State

Parameter	OR	95% CI^b^	
SP dosage			
0	1.14	0.79	1.64
1	2.04*	1.37	3.02
2	1.21	0.83	1.76
3+ (ref)			
Intervention			
Yes	1.10	0.79	1.53
No (ref)			
At least 1 ANC visit			
Yes	0.57*	0.43	0.75
No (ref)			
Primigravida^a^			
Yes	1.87*	1.38	2.53
No (ref)			
Place of birth			
Facility	1.86*	1.23	2.80
Home (ref)			
Month of birth			
July–November	0.85	0.64	1.13
April–June (ref)			

9453 births that occurred >28 weeks gestation

^a^n = 9438

^b,^* Significant at p < 0.05

Table 12 shows the adjusted odds of a woman having a stillbirth. Fewer doses of SP taken was associated with higher odds of stillbirth; only the difference between the first dose and three doses was significant at p < 0.05. (adj. OR 1.77). As in the univariate results, there was no significant association between residing in the intervention (vs. counterfactual) sites and the odds of stillbirth, nor with giving birth later on in the course of the study. Adjusting for all of the other variables in the model, any ANC attendance was associated with a statistically significant 66% decline in the odds of a woman having a stillbirth (adj. OR 0.44). Definitively, primigravida women (adj. OR 1.81) had higher odds of a stillbirth, relative to multigravida women. Women that gave birth in a facility (adj. OR 1.99) were significantly more likely to have had a stillbirth, relative to those who did not give birth in a facility.

**Table 12:** Adjusted odds of having a stillbirth (vs. a live birth) between April and November 2015 in four LGA’s in Sokoto State

Parameter	OR	95% CI	
SP dosage			
0	0.76	0.49	1.18
1	1.77	1.18	2.66
2	1.18	0.80	1.74
3+ (ref)			
Intervention			
Yes	0.90	0.62	1.30
No (ref)			
At least 1 ANC visit			
Yes	0.44	0.32	0.61
No (ref)			
Primigravida			
Yes	1.81	1.33	2.47
No (ref)			
Place of birth			
Facility	1.99	1.28	3.10
Home (ref)			
Month of birth			
July–November	0.81	0.60	1.11
April–June (ref)			

*n = 9438; L-R *p* value < 0.001; c-statistic = 0.65; Hosmer and Lemeshow Goodness-of-Fit pr Chi-sq >0.3753 test

As with head circumference, ANC attendance and higher SP dosages were significantly associated with the odds of stillbirth. None of the tests for any interaction between ANC attendance and SP dosages were either significant or added importance to the model fit. As with head circumference findings, the impact of higher SP dosages and ANC attendance with the odds of stillbirth appeared to operate independently.

### Findings relating to costing

#### Cost per woman

Given the total number of women reached in this study with SP, the average cost per woman who received SP1-3 ranged from N1,132 ($5.7) to N1,518 ($7.6) in the three intervention LGA to N5,326 ($26.7) in the counterfactual LGA (see Table 13). Given that majority of the costs associated with the delivery of SP doses per woman were fixed costs and are, therefore, comparable across LGAs in the intervention and counterfactual alike, the spread in cost per woman reached with SP1-3 in intervention LGAs is explained primarily by differences in the actual number of women that got all three doses of SP. A higher number of women who got all three doses is a function of reaching the most women with SP1 in the first instance and subsequently ensuring that there were few dropouts in women who received SP2 and SP3. The higher cost per woman in the counterfactual LGA reflects the overall low number of women on SPI, the higher dropout by SP2 and the absence of women reached with SP3. The 3.5- to 4.6-fold lower average costs per woman in the intervention LGA suggests that strategies that substantially increase coverage and significantly reduce drop out in-between doses, will lower the average cost per woman.

**Table 13:** Cost per woman who received SP 1-6 contrasted by intervention and counterfactual LGAs

	LGAs			
	Goronyo [Cost per woman in 2015 Nigerian Naira (NGN)]	Silame [Cost per woman in 2015 Nigerian Naira (NGN)]	Dange Shuni [Cost per woman in 2015 Nigerian Naira (NGN)]	Yabo [Cost per woman in 2015 Nigerian Naira (NGN)]
SP dose level				
Women who received SP1	723	1228	947	4155
Women who received SP2	963	1575	1235	6498
Women who received SP3	1710	2857	2371	
Women who received SP4+ (SP5, SP6)	4889	9269	12,169	
Average cost per woman SP1–SP3	1132	1887	1518	5326
Average cost per woman SP1–SP4+	2071	3732	4180	5326

#### Cost per SP dose

The estimated cost per course per dose of SP1, SP2 and SP3 ranged from NGN 185 ($0.9) to NGN288 ($1.45) in the intervention LGAs compared to a much higher cost of NGN 1382 ($6.98) in the counterfactual LGA. The estimated cost per course per dose of SP1, SP2, SP3 and SP4+ ranged from NGN 239 ($1.21) to NGN373 ($1.89) in the intervention LGAs, compared to NGN 1843 ($9.21) in the counterfactual LGA (see Table 14). The observed substantial difference in the cost of a SP1-3 regimen in the counterfactual LGA is attributable to no reportable distribution activity with SP3 and higher. The incremental cost per dose of SP1, SP2, SP3 and SP4+ on SP1, SP2, SP3 and ranged from NGN 53.5 ($0.27) to NGN85 ($0.43) in the intervention LGAs compared to a much higher NGN 460.87 ($2.33) in the counterfactual. The observed five- to eightfold higher cost per SP dose in the counterfactual LGA is the cost of underperformance of not being at programme scale.

**Table 14:** Estimated cost per dose of SP

	LGA			
	Intervention			Counterfactual
	Goronyo [Costs and cost per SP dose in 2015 Nigerian Naira (NGN)]	Silame [Costs and cost per SP dose in 2015 Nigerian Naira (NGN)]	Dange Shuni [Costs and cost per SP dose in 2015 Nigerian Naira (NGN)]	Yabo [Costs and cost per SP dose in 2015 Nigerian Naira (NGN)]
SP dose				
Women who received SP1	1,637,746	1,515,582	1,453,361	1,286,860
Women who received SP2	1,637,926	1,515,865	1,453,586	1,288,425
Women who received SP3	1,639,192	1,517,808	1,455,498	1,288,425
Women who received SP4+ (SP5, SP6)	1,646,255	1,530,833	1,476,856	1,288,425
Total cost of SP1–SP3	4,914,866	4,549,256	4,362,447	3,863,712
Total cost of SP1–SP4+	6,561,121	6,080,088	5,839,303	5,152,137
Total number of women who received any SP1, SP2 and SP3 doses	22,760	13,391	15,424	2294
Total number of women who received any SP1, SP2, SP3 and SP4+ doses	23,578	13,797	15,664	2294
Cost per SP1,SP2 and SP3 doses	216	340	283	1684
Cost per SP1, SP2, SP3 and SP4+ doses	278	441	373	2246

## Discussion

To the best of our knowledge, this is the first published implementation research study that has prospectively examined the association between SP use and newborn outcomes, at scale, in community settings. This study is the largest cohort of pregnant women included in a study designed and powered to examine the effects of IPTp-SP delivered at scale.

Study data show that IPTp-SP can be delivered safely to pregnant mothers, in accordance with Nigeria’s Federal Ministry of Health and applicable WHO guidelines. The results have also shown that community-based distribution of SP, twinned with facility-based distribution, produced a superior and sustained coverage in the use of SP by pregnant women, at scale. In this context, community-based distribution of SP substantially increased facility-based distribution of SP. Critical attention given to realizing authentic community ownership, through upfront education and an early community discernment of SP’s beneficial results, contributed to a dramatic increase in the consumption of SP.

The MiPP project did not directly or indirectly intervene on the availability of water to ingest medicines in facilities, although this is a factor that has been cited as a contributor to low IPTp-SP coverage [36–38]. The surge of IPTp-SP use in facilities in the intervention LGAs suggests that increasing demand for IPTP-SP would intrinsically motivate to overcome barriers to accessing water to ingest her medicine. Additionally, in the intervention LGA’s, IPTp-SP was recorded only after it had been observed ingested, by the CBV.

Any antenatal care attendance was strongly predictive of larger newborn head circumference and a reduced odds of stillbirths. The authors call for urgent action by policy makers and programmes to increase communities’ access to and utilization of quality ANC services in Sokoto State and Nigeria. This finding affirms the Global Malaria in Pregnancy Working Group’s call that any efforts intended to increase IPTp-SP delivery at scale, need to also increase ANC usage. The study reinforced, but was not conclusive about, the increased susceptibility of primigravid mothers to having stillbirths. There is already well-established local, tacit knowledge in Sokoto State communities that primigravid mothers suffer greater risks in pregnancy, and therefore need more care. It is recommended that advocacy and community education efforts capitalize on this notion to increase greater use of ANC and health facility delivery services by primigravid women. The study also confirmed a well-established finding that male newborns had larger head circumferences than female ones, and lends biological validity the study findings as a whole.

The finding that women who gave birth in a health facility compared to home deliveries had higher odds of having stillborn babies was puzzling at first glance. However, in a setting like Sokoto State where home delivery is regretfully almost universal, women with complications are more likely to be referred to primary health facilities, and consequently with their newborns suffer higher case fatalities than home births. The implication is that primary health facilities operate as de facto secondary level care facilities, without requisite support. More needs to be done to increase health centres’ preparedness to deliver basic emergency obstetric care. Policy actions that remove barriers to women’s access health facilities for deliveries in Sokoto State and Nigeria are urgently warranted.

Given these findings on the benefits of SP3 doses and higher on newborn survival, the pathway to realize population level benefits rests with getting IPTp-SP interventions implemented at scale. The evidence suggests that a community distribution stratagem, that is well designed to increase coverage, and also amplifies the use of facility-based services, is both feasible and indispensable in Sokoto State and Nigeria. This programme used a health systems approach combined with human-centered design to build on the Nigeria National Primary Health Care Development Agency’s “minimum health care package guidelines”. Lessons from this programme can be used by state governments who would like to accelerate at scale implementation of primary health care service.

SP was readily integrated to other ongoing MNCH activities including the provision of chlorhexidine gel for care of the newborn cord and misoprostol to prevent postpartum haemorrhage. It is also instructive that there were effectively no incidents of stock out of SP of note during the study; this was instrumental to the results. It was helpful that a community health information system, derived from household databases aided the proactive identification of women who received reminders. It enabled timeliness and greater precision in reaching women with SP services. Presently, the Federal Ministry of Health’s web-based health information system, DHIS2 does not collect SP3 or higher doses at all. The DHIS2 should be revised to capture at least three doses of SP, and higher, in line with current national and WHO SP policy guidelines.

Another factor that may have facilitated the scale up of IPTp-SP delivery, was the power of social diffusion. Women who had received SP gave public testimony that they felt their babies at birth, were larger than in previous births; they made uncharacteristic, prideful public displays of their babies. Mothers also gave personal testimonies to their peers that SP made them feel healthier while being pregnant. This phenomenon may have helped to increase the proportion of women who declared their pregnancy earlier, started SP use earlier, and received higher mean doses of SP. It should be emphasized that mothers and communities want to experience tangible results, as an incentive to lend critical support to scaled-up health interventions. With acceptance of the promise of results, communities, as a measure of their trust, used their own funds to recruit additional volunteers to cover hard-to-reach areas. They were also very proactive in the use of information from monthly review meetings to sustain programme momentum.

Inclusive of the cost of SP, it cost between US$0.93 and $1.20 per three doses in the intervention LGAs. These lower cost ratios were the result of increased demand for SP, enabled by community-based distribution plus and facility distribution, as well as the reduced number missed opportunities in clinics. The higher equivalent cost of $6.9 per three doses in the counterfactual LGA, reflects the higher cost associated with a facility-only stratagem of distribution and the ensuing low demand for SP. A scale-up of IPTp-SP that utilizes both community and facility based service delivery, will reduce the cost per woman served and cost per dose given; it is, therefore, recommended in Sokoto State and in Nigeria.

The finding of a decline in the mean newborn head circumference in births month on month from April to November in both intervention and counterfactual groups, suggest factors other than placental malaria at play in worsening newborn outcomes. One likely factor, beyond the scope of this study, is the role of maternal nutrition on newborn outcomes. Mothers whose babies were born in April had more access to post harvest foods for at least 5 months. A combination of better maternal nutrition and better protection from placental malaria is likely to increase the odds of newborn survival. The interplay of maternal nutrition and placental malaria is an area of further study.

### Limitations

The data collection instrument in this study was designed to collect a handful of simple metrics that could evaluate the impact of community based IPTp-SP in a context of low literacy. Data collected by CBHV supervisors did not capture many of the socioeconomic, nutritional and behavioral issues that affect newborn outcomes.

The WHO defines a stillbirth as a pregnancy as “*a baby born with no signs of life at or after 28* *weeks’ gestation*” [39]. The definition of stillbirth used in this study was a crude, yet pragmatic, measure in the community programme context of Sokoto State. While this does present a potential for misclassification of stillbirths, the finding of biologically plausible associations between SP doses and a reduction in the odds of stillbirths, enhance the internal validity of the study.

Although every effort was undertaken to standardize head circumference measurements, it is probable that there were variations in where the tape measure was placed. Given the large sample size of newborns, errors in head circumference measurements would be normally distributed. Every effort was made to ensure head circumference measurement as close to birth and no later than 7 days postpartum. On this basis, as mentioned earlier, 2521 newborns principally in the intervention group, that were 8 days or older before they could be measured were excluded. The overall validity of the study is strengthened by findings that confirm an already established, biologically plausible, association between gestational age and the sex of the newborn on newborn head circumference.

The intervention deployed, although primarily the introduction of SP at the community and the training of facility-based health workers to reduce missed opportunities, it also included the restoration of linkages between communities and facilities, to promote referrals. The system for finding and enumerating women, which doubled as community mobilization, and was confined to intervention LGAs, and not introduced into the counterfactual LGA. To have introduced the house-to-house enumeration visits in the counterfactual LGA, would have increased community awareness and increased demand for SP, as was evident in the spike in SP use that occurred with the data collection on birth outcomes. In effect, the house-to-house enumeration served as a co-intervention. As seen in Table 4, the house-to-house enumeration, in two intervention areas, showed a higher number of eligible women than were estimated from official population estimates. This suggests that, if anything, there is most probably have an under estimate of the number of eligible women in the counterfactual area. If this was the case, coverage rates of IPTp-SP in the counterfactual LGA would be even lower than estimated.

## Conclusion

The study findings underscore the untapped potential of results-driven primary health care programming inclusive of trained community health workers, to accelerate and safely implement IPTp-SP delivery at scale. The scale up of IPTp-SP programmes, that include a vital community distribution component, is an important stratagem for inclusion in national policy strategies. An optimally scaled-up IPTp-SP programme is more likely to reach the underserved, also the most likely to suffer inequities. Consideration should be given to systems thinking enabled human-centered design principles to help match scale up objectives with strategies most likely to yield local trust, ownership and predictable availability of quality services.

## Additional files

**Additional file 1.** Data collection form for Outcomes in the Intervention and Counterfactual LGA’s.

**Additional file 2.** Sample of Pictograms, on the back of color-coded SP administration cards, designed by CBHV supervisors.

**Additional file 3.** Colour-coded dose cards for SP distribution.

**Additional file 4.** Unadjusted mean Head Circumference (HC) in millimeters (mm) among live newborns born between April and November 2015 (without October), and differences in mean HC by available variables.

**Additional file 5.** Adjusted Associations between selected variables and the mean head circumference (HC) of newborns, between April and November 2015 (without October).

## Abbreviations

ACT: artemisinin combination therapy
AFRO: World Health Organization Africa region
ANC: antenatal care
CBHV: community based health volunteers
DHIS2: District Health Information System 2
DOT: directly observed therapy
HC: newborn head circumference
HRB: household register book
HRS: household registration system
IPTp: intermittent preventive treatment in pregnancy
IPTp-SP: intermittent preventive treatment in pregnancy with sulphadoxine-pyrimethamine
ITN: insecticide treated nets
LLIN: long lasting insecticide treated nets
JSI: JSI Research & Training Institute, Inc
LGA: local government area
LMP: last menstrual period
M&E: monitoring and evaluation
MiP: malaria in pregnancy
MiPP: malaria in pregnancy project
MSF: monthly summary form
RBM: roll back malaria partnership
RDT: rapid diagnostic testing kits
SBR: stillbirth rate
SMOH: Sokoto State Ministry of Health
TSHIP: Targeted States High Impact Project
USAID: United States Agency for International Development
WDC: Ward Development Committee
WHO: World Health Organization
