# Supplementary material for: Scaling-up the use of sulfadoxine-pyrimethamine for the preventive treatment of malaria in pregnancy: results and lessons on scalability, costs and programme impact from three local government areas in Sokoto State, Nigeria
Source: Malar J. 2016 Nov 4;15:533. doi: 10.1186/s12936-016-1578-x (PMC5097385; doi:10.1186/s12936-016-1578-x)
Supplement: Supplementary file 1 — Additional file 1. Data collection form for Outcomes in the Intervention and Counterfactual LGA’s. [file 12936_2016_1578_MOESM1_ESM.docx]

| **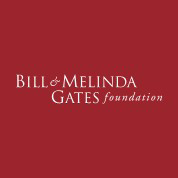 SOKOTO STATE MINISTRY OF HEALTH 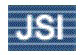**  **IN COLLABORATION WITH BILL & MELINDA GATE FOUNDATION**  **(MALARIA IN PREGNANCY PROJECT)**  **IMMEDIATE PREGNANCY OUTCOME FORM**  **INTERVENTION LGA**  **1.INFORMATION ON PARENTS** | | | | | | | | | | | | | | | | | | | | | | | | | | | | | | | |  | |  |
| --- | --- | --- | --- | --- | --- | --- | --- | --- | --- | --- | --- | --- | --- | --- | --- | --- | --- | --- | --- | --- | --- | --- | --- | --- | --- | --- | --- | --- | --- | --- | --- | --- | --- | --- |
| 1.1. Compound ID………………………… | | | | | | | |  | | |  | | |  | | | | | |  | | |  | | |  | |  | |  | | | COMP_ID | |
| 1.2. Household Name and ID--------------------------------------------------------------- | | | | | | | | | | | | | | | | | | | | | | | | | | |  | | |  | | | HH_ID |  |
| 1.3. Permanent ID of Mother | |  | |  |  | |  | | |  | | |  | | | |  |  | | |  | | | |  | |  | | |  | | | MOTHER_ID |  |
| 1.4 Name of Mother _____________________________________________________________ | | | | | | | | | | | | | | | | | | | | | | | | | | | | | | | | |  |  |
| 1.5. Date of delivery ……………….………………… | | | | | | | | |  | | |  | | | |  | | |  | | |  | | |  | | | |  | |  | | BIRTHDATE |  |
| 1**.6**. Place of Birth | 1. Hospital | | 2.Health Centre/Post/Clinic | | | | | | | | | | | | 3. Home | | | | | | | | | 4.Traditional Birth  Attendant’s home | | | | | | | | | P_BIRTH |  |
|  | 5. Other ( Specify):--------------------------------------------------------------- | | | | | | | | | | | | | | | | | | | | | | | | | | | | | | | |  |  |
| 1.7 Did you go for antenatal care during this pregnancy? | | | | | | 1. YES 2. NO | | | | | | | | | | | | | | | | | | | | | | | | | | | ANC_ATT |  |
| 1.8 If yes, where did you go for antenatal clinic? | | | | | | PHC…………………………………...……………..1  GENERAL HOSPITAL……………………………..2  DISPENSARY……………………………………….3  OTHERS (SPECIFY)…….…………………………..4 | | | | | | | | | | | | | | | | | | | | | | | | | | | P_ANC |  |
| 1.9 How many times did you attend antenatal care? | | | | | | NUMBER OF VISITS ………………….. | | | | | | | | | | | | | | | | | | | | | | | | | | | NUM_ANC |  |

| 1.10 Outcome of the pregnancy…………... | 1. Live birth | 2. Still birth | 3. Miscarriage | 4. Abortion | PREG_OUT |
| --- | --- | --- | --- | --- | --- |

| 1**.11**. What was used in cutting the umbilical cord? | 1. New razor blade | | 2. Old razor blade | | 3. Knife | | CUTCOD |
| --- | --- | --- | --- | --- | --- | --- | --- |
|  | 4. Nothing | 5. Scissors | | 6. Other (Specify)  ---------------------- | | 8. Don’t Know |  |

| 1**.12** What was applied to the umbilical after it was cut? | 1. Chlorhexidine | 2. Heat Application/Vaseline ointment | | 3. Warm Conference/Vaseline ointment | | CORDRESS |
| --- | --- | --- | --- | --- | --- | --- |
|  | 4. Methylated Spirit | 5. Nothing | 6. Other (specify)  ------------------------ | | 7. Don’t Know |  |

**ANSWER 1.13 IF 1.12 IS 1 (Chlorhexidine)**

| 1.13 Were there any side effects after applying chlorhexidine gel to the baby’s umbilical cord? | | Yes……………………..……………………….…..1  No……………………….…..………..……………..2  Don’t remember………………..…….………….…8  Not Applicable……………………….……….……9 | | | | | | | IF 2 OR 8 GOTO 2.1 | | | | | | | | SIDE_EFFT1 | | | | | | |  |  |
| --- | --- | --- | --- | --- | --- | --- | --- | --- | --- | --- | --- | --- | --- | --- | --- | --- | --- | --- | --- | --- | --- | --- | --- | --- | --- |
| 1.14. If yes above, which one?  **(CIRCLE ALL THAT APPLY)** | | Redness………..…….….………….……..………..Yes No  Skin rashes…………..……………..………..….….Yes No  Discharge….………….……………….………..….Yes No  Swelling………………………….…….………......Yes No  Others(specify)…………………………....……….Yes No | | | | | | | | | | | | | | | SIDE_EFFT2 | | | | | | |  |  |
| **2. MOTHER’S BIRTH RECORD HISTORY:** | | | | | | | | | | | | | | | | | | | | | | | | | |
| 2.1. Is this your first delivery?……………………………………..………………. | | | | | | | | 1. Yes | | | | | | | 2. No | | | | | | FIRST_DEL | | | | |
| **Please enter “88” for 2.2 if answer to 2.1 is 1.Yes.** | | | | |  |  | |  | | | |  | |  | |  | | |  | | |  |  | | |
| 2.2. How many deliveries have you had, including this child?................................................ | | | | | | | | | | | | |  | | |  | | | | CEB | | | | | |
| 2.2. How many came out alive?............................................................................................... | | | | | | | | | | | | |  | | |  | | | | ALIVE | | | | | |
| 2.3. Total number of babies delivered in this pregnancy:…………………    2.4. Number of new born alive from this pregnancy:…………………………  2.5. At what month of pregnancy did you deliver you baby/babies?:………… | | | |  |  |  |  | |  | |  | |  | | |  | | | | TBIRTH  LBIRTH  GA_MTH | | | | | |
|  |  |  |  |  |  |  |  |  |  |  |  |  |  |  |  |  | | | |  |  |  |  |  |  |
|  |  |  |  |  |  |  |  |  |  |  |  |  |  | | |  | | | |  |  |  |  |  |  |
| \| **2.6** \| Did you take misoprostol?................................... \| Yes……………………………..1  No………………………….…..2 \| \| IF 2 GOTO 2.9 \| \| \| --- \| --- \| --- \| --- \| --- \| --- \| \| **2.7** \| Did you experience any side effects after taking misoprostol?.......................................................... \| Yes….………………………….1  No……………………………...2 \| \| IF 2 OR 8 GOTO 2.9 \| \| \| **2.8** \| If yes, which one? …………………….………..  **(CIRCLE ALL THAT APPLY)** \| Shivering…………………..……….  Nausea……………………..……….  Abdominal cramping……………….  Vomiting…………………...............  Diarrhea……………………..……...  Fever ……………………………….  Others(specify)…..………………… \| \| Yes  1  1  1  1  1  1 \| No  2  2  2  2  2  2 \| \| **2.9** \| During this pregnancy did you take SP to prevent you from getting malaria? \| Yes……………………………..1  No………………………….…..2 \| \| IF 2 GOTO 2.12 \| \| \| **2.10** \| If yes, How many times did you take SP during this pregnancy…………………………… \| \| \|  \|  \| \| **2.11** \| Do you have LLIN? \| Yes……………………………..1  No………………………….…..2 \| \| IF 2 GOTO 3.1a \| \| \| **2.12** \| Are you sleeping under LLIN? \| Yes……………………………..1  No………………………….…..2 \| \| IF 2 GOTO 3.1a \| \| \| **2.13** \| How often do you sleep under LLIN? \| 1. Daily \| 2. Frequently \| \| \|   T_MISO  EXP_MIS  M_EFF  SP  SP_NUM  HAV_NET  SLP_NET  SLP_TYP **INFORMATION ON CHILD/CHILDREN:** | | | | | | | | | | | | | | | | | | | | | | | | | |
| 3.1a Name of child __________________________________________________________________________ | | | | | | | | | | | | | | | | | | | | | | | | | |

| 3.2a Sex:………………………………………… | 1=MALE | | | 2=FEMALE | SEX1 |
| --- | --- | --- | --- | --- | --- |
| 3.3a Head circumference in centimeter …………………….. |  |  |  |  | HC1 |
|  | | | | | |
| 3.2a Sex:………………………………………… | 1=MALE | | | 2=FEMALE | SEX2 |
| 3.3a Head circumference in centimeter …………………….. |  |  |  |  | HC22 |
|  | | | | | |

CBHV’s Supervisors Name and Date of Visit…………………………………………….

| **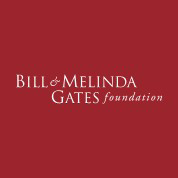 SOKOTO STATE MINISTRY OF HEALTH 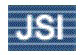**  **IN COLLABORATION WITH BILL & MELINDA GATE FOUNDATION**  **(MALARIA IN PREGNANCY PROJECT)**  **IMMEDIATE PREGNANCY OUTCOME FORM**  **COUNTERFACTUAL LGA**  1.1a Ward Name:…………………………………………………………………………………………… | | | | | | | | | | | | | | WARD_Name | |
| --- | --- | --- | --- | --- | --- | --- | --- | --- | --- | --- | --- | --- | --- | --- | --- |
| 1.1b Settlement Name:……………………………………………………………………………………… | | | | | | | | | | | | | | SETTL_Name | |
| 1.1c Compound Name………………………………………………………………………………………. | | | | | | | | | | | | | | COMP_Name | |
| 1.2. Household Head Name:………………………………………………………………………………… | | | | | | | | | | | | | | HHH_Name |  |
| 1.3 Name of Mother _____________________________________________________________ | | | | | | | | | | | | | |  |  |
| 1.5. Date of delivery ……………….………………… | | | |  |  | |  |  |  | |  |  |  | BIRTHDATE |  |
| 1**.6**. Place of Birth | 1. Hospital | 2.Health Centre/Post/Clinic | | | | 3. Home | | | | 4.Traditional Birth  Attendant’s home | | | | P_BIRTH |  |
|  | 5. Other ( Specify):--------------------------------------------------------------- | | | | | | | | | | | | |  |  |
| 1.7 Did you go for antenatal care during this pregnancy? | | | 1. YES 2. NO | | | | | | | | | | | ANC_ATT |  |
| 1.8 If yes, where did you go for antenatal clinic? | | | PHC…………………………………...……………..1  GENERAL HOSPITAL……………………………..2  DISPENSARY……………………………………….3  OTHERS (SPECIFY)…….…………………………..4 | | | | | | | | | | | P_ANC |  |
| 1.9 How many times did you attend antenatal care? | | | NUMBER OF VISITS ………………….. | | | | | | | | | | | NUM_ANC |  |

| 1.10 Outcome of the pregnancy…………... | 1. Live birth | 2. Still birth | 3. Miscarriage | 4. Abortion | PREG_OUT |
| --- | --- | --- | --- | --- | --- |

| 1**.11**. What was used in cutting the umbilical cord? | 1. New razor blade | | 2. Old razor blade | | 3. Knife | | CUTCOD |
| --- | --- | --- | --- | --- | --- | --- | --- |
|  | 4. Nothing | 5. Scissors | | 6. Other (Specify)  ---------------------- | | 8. Don’t Know |  |

| 1**.12** What was applied to the umbilical after it was cut? | 1. Chlorhexidine | 2. Heat Application/Vaseline ointment | | 3. Warm Conference/Vaseline ointment | | CORDRESS |
| --- | --- | --- | --- | --- | --- | --- |
|  | 4. Methylated Spirit | 5. Nothing | 6. Other (specify)  ------------------------ | | 7. Don’t Know |  |

**ANSWER 1.13 IF 1.12 IS 1 (Chlorhexidine)**

| 1.13 Were there any side effects after applying chlorhexidine gel to the baby’s umbilical cord? | Yes……………………..……………………….…..1  No……………………….…..………..……………..2  Don’t remember………………..…….………….…8  Not Applicable……………………….……….……9 | IF 2 OR 8 GOTO 2.1 | SIDE_EFFT1 |
| --- | --- | --- | --- |

| 1.14. If yes above, which one?  **(CIRCLE ALL THAT APPLY)** | Redness………..…….….………….……..………..Yes No  Skin rashes…………..……………..………..….….Yes No  Discharge….………….……………….………..….Yes No  Swelling………………………….…….………......Yes No  Others(specify)…………………………....……….Yes No | | | | | | | | | | | | | SIDE_EFFT2 | | | | | |  |
| --- | --- | --- | --- | --- | --- | --- | --- | --- | --- | --- | --- | --- | --- | --- | --- | --- | --- | --- | --- | --- |
| **2. MOTHER’S BIRTH RECORD HISTORY:** | | | | | | | | | | | | | | | | | | | | |
| 2.1. Is this your first delivery?……………………………………..………………. | | | | | | 1. Yes | | | | | | 2. No | | | | | FIRST_DEL | | | |
| **Please enter “88” for 2.2 if answer to 2.1 is 1.Yes.** | | |  |  | |  | | |  | |  | |  | |  | | |  |  | |
| 2.2. How many deliveries have you had, including this child?................................................ | | | | | | | | | |  | | |  | | | CEB | | | | |
| 2.2. How many came out alive?............................................................................................... | | | | | | | | | |  | | |  | | | ALIVE | | | | |
| 2.3. Total number of babies delivered in this pregnancy:…………………    2.4. Number of new born alive from this pregnancy:…………………………  2.5. At what month of pregnancy did you deliver you baby/babies?:………… | |  |  |  |  | |  |  | |  | | |  | | | TBIRTH  LBIRTH  GA_MTH | | | | |
|  |  |  |  |  |  |  |  |  |  |  |  |  |  | | |  |  |  |  |  |
|  |  |  |  |  |  |  |  |  |  |  | | |  | | |  |  |  |  |  |
| \| **2.6** \| Did you take misoprostol?................................... \| Yes……………………………..1  No………………………….…..2 \| \| IF 2 GOTO 2.9 \| \| \| --- \| --- \| --- \| --- \| --- \| --- \| \| **2.7** \| Did you experience any side effects after taking misoprostol?.......................................................... \| Yes….………………………….1  No……………………………...2 \| \| IF 2 OR 8 GOTO 2.9 \| \| \| **2.8** \| If yes, which one? …………………….………..  **(CIRCLE ALL THAT APPLY)** \| Shivering…………………..……….  Nausea……………………..……….  Abdominal cramping……………….  Vomiting…………………...............  Diarrhea……………………..……...  Fever ……………………………….  Others(specify)…..………………… \| \| Yes  1  1  1  1  1  1 \| No  2  2  2  2  2  2 \| \| **2.9** \| During this pregnancy did you take SP to prevent you from getting malaria? \| Yes……………………………..1  No………………………….…..2 \| \| IF 2 GOTO 2.12 \| \| \| **2.10** \| If yes, How many times did you take SP during this pregnancy…………………………… \| \| \|  \|  \| \| **2.11** \| Do you have LLIN? \| Yes……………………………..1  No………………………….…..2 \| \| IF 2 GOTO 3.1a \| \| \| **2.12** \| Are you sleeping under LLIN? \| Yes……………………………..1  No………………………….…..2 \| \| IF 2 GOTO 3.1a \| \| \| **2.13** \| How often do you sleep under LLIN? \| 1. Daily \| 2. Frequently \| \| \|   T_MISO  EXP_MIS  M_EFF  SP  SP_NUM  HAV_NET  SLP_NET  SLP_TYP **INFORMATION ON CHILD/CHILDREN:** | | | | | | | | | | | | | | | | | | | | |
| 3.1a Name of child __________________________________________________________________________ | | | | | | | | | | | | | | | | | | | | |

| 3.2a Sex:………………………………………… | 1=MALE | | | 2=FEMALE | SEX1 |
| --- | --- | --- | --- | --- | --- |
| 3.3a Head circumference in centimeter …………………….. |  |  |  |  | HC1 |
|  | | | | | |
| 3.2a Sex:………………………………………… | 1=MALE | | | 2=FEMALE | SEX2 |
| 3.3a Head circumference in centimeter …………………….. |  |  |  |  | HC22 |
|  | | | | | |

Home Visitors Name and Date of Visit…………………………………………….
