# Supplementary material for: Scaling-up the use of sulfadoxine-pyrimethamine for the preventive treatment of malaria in pregnancy: results and lessons on scalability, costs and programme impact from three local government areas in Sokoto State, Nigeria
Source: Malar J. 2016 Nov 4;15:533. doi: 10.1186/s12936-016-1578-x (PMC5097385; doi:10.1186/s12936-016-1578-x)
Supplement: Supplementary file 2 — Additional file 2. Sample of Pictograms, on the back of color-coded SP administration cards, designed by CBHV supervisors. [file 12936_2016_1578_MOESM2_ESM.docx]

**Supplemental File 2: The Sample of Pictograms on the back of color coded SP administration cards designed by CBHV supervisors. This was used to ease identification of households of pregnant women due for SP by CBHVs who are unable to read and write.**

1.
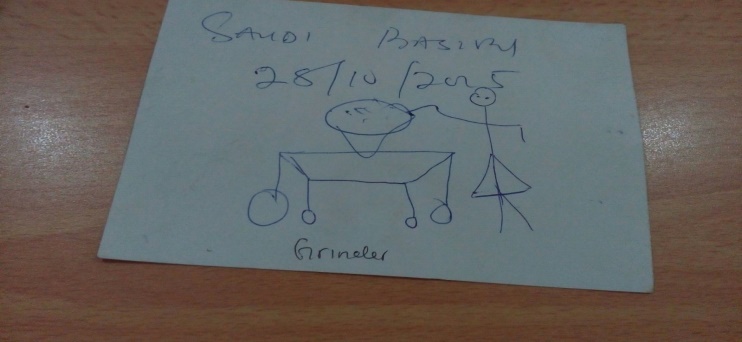


SP administration card for a woman who owns a grinding machine

1.
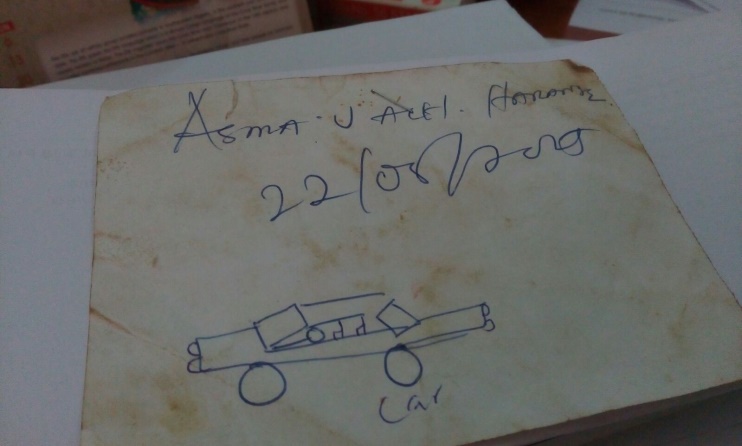


SP administration card for a pregnant woman whose husband owns a car

1.
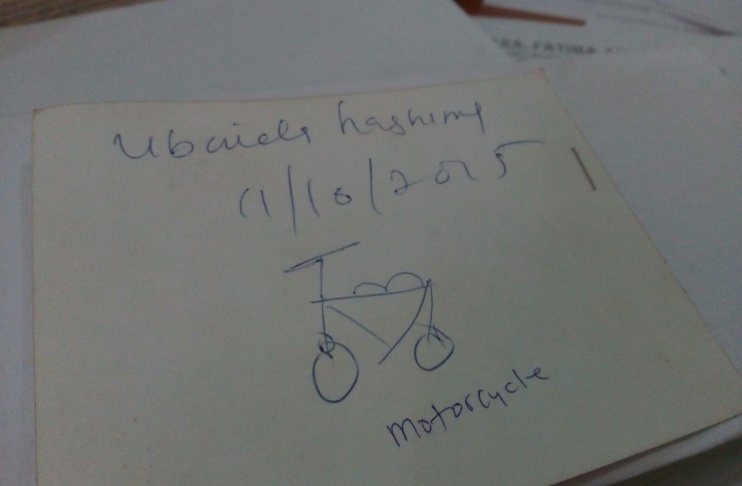


SP administration card for a pregnant woman whose husband owns a motorcycle

1.
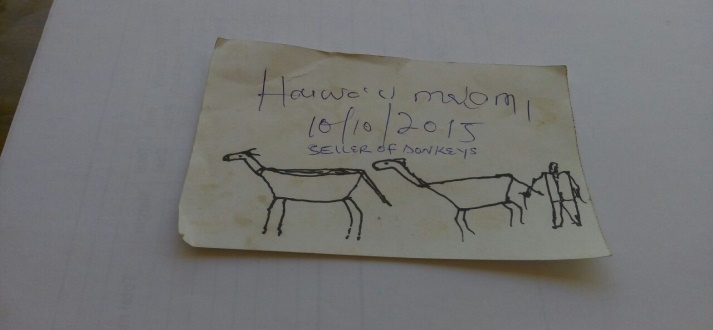


SP administration card of a pregnant woman whose husband is a donkey seller

1.
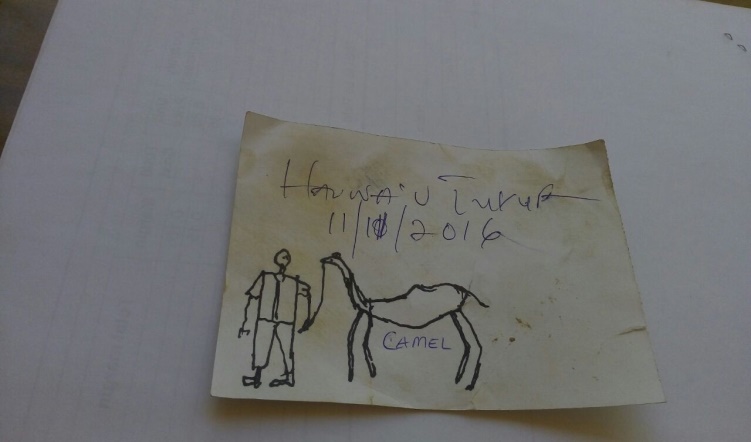


SP administration card of a pregnant woman whose husband owns a camel

1.
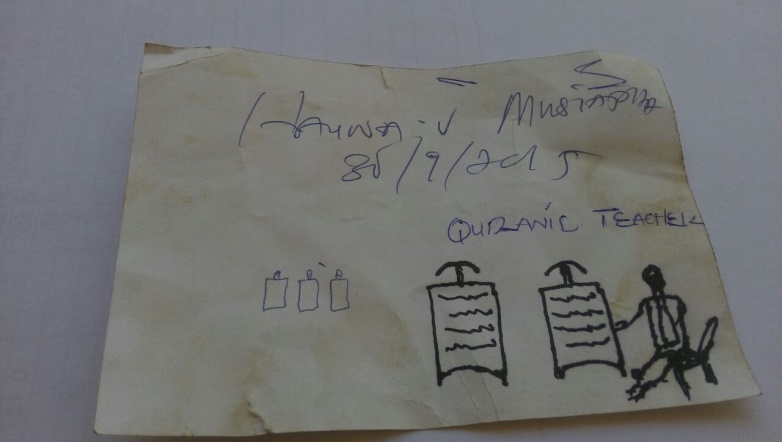


SP administration card of a pregnant woman whose husband is a Quranic school teacher
