# Supplementary material for: Scaling-up the use of sulfadoxine-pyrimethamine for the preventive treatment of malaria in pregnancy: results and lessons on scalability, costs and programme impact from three local government areas in Sokoto State, Nigeria
Source: Malar J. 2016 Nov 4;15:533. doi: 10.1186/s12936-016-1578-x (PMC5097385; doi:10.1186/s12936-016-1578-x)
Supplement: Supplementary file 3 — Additional file 3. Colour-coded dose cards for SP distribution. [file 12936_2016_1578_MOESM3_ESM.docx]

| Supplementary File 3: Color coded dose cards for SP distribution | | | | | | | | | | | | | | | | | | | | | | |
| --- | --- | --- | --- | --- | --- | --- | --- | --- | --- | --- | --- | --- | --- | --- | --- | --- | --- | --- | --- | --- | --- | --- |
| CBHV Household  Sulphadoxine –Pyrimethamine (SP) Administration  Card for Pregnant Women  **1^st^ DOSE**  Compound Number | | | | | | | | | | | 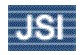  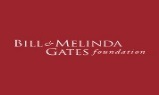  CBHV Household Sulphadoxine – Pyrimethamine (SP)  Administration Card for Pregnant Women  **1^st^ DOSE** | | | | | | | | | | | |
|  | | | | | | | | | | |  | | | | | | | | | | | |
| Household & Woman ID |  |  | |  | |  |  | |  |  |  |  |  |  |  |  |  |  |  |  |  |  |
|  | | | | | | | | | | |  |  |  |  |  |  |  |  |  |  |  |  |
|  | | |  | |  | | |  | | | Compound Number | | | | | | | | Household & Woman ID | | | |
|  | | | | | | | | | | |  |  |  |  |  |  |  |  |  |  |  |  |

| CBHV Household  Sulphadoxine –Pyrimethamine (SP) Administration  Card for Pregnant Women  **2^nd^ DOSE**  Compound Number | | | | | | | | | | | 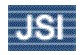  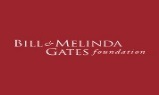  CBHV Household Sulphadoxine – Pyrimethamine (SP)  Administration Card for Pregnant Women  **2^nd^ DOSE** | | | | | | | | | | | |
| --- | --- | --- | --- | --- | --- | --- | --- | --- | --- | --- | --- | --- | --- | --- | --- | --- | --- | --- | --- | --- | --- | --- |
|  |  |  | |  | |  |  | |  |  |  |  |  |  |  |  |  |  |  |  |  |  |
| Household & Woman ID | | | | | | | | | | |  |  |  |  |  |  |  |  |  |  |  |  |
|  | | |  | |  | | |  | | | Compound Number | | | | | | | | Household & Woman ID | | | |
|  | | | | | | | | | | |  |  |  |  |  |  |  |  |  |  |  |  |

| CBHV Household  Sulphadoxine –Pyrimethamine (SP) Administration  Card for Pregnant Women  **3^rd^ DOSE and Referral to Health Facility for 4^th^ DOSE PLUS**  Compound Number | | | | | | | | | | | 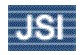  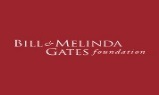  CBHV Household Sulphadoxine – Pyrimethamine (SP)  Administration Card for Pregnant Women  **3^rd^ DOSE and Referral to Health Facility for 4^th^ DOSE PLUS** | | | | | | | | | | | |
| --- | --- | --- | --- | --- | --- | --- | --- | --- | --- | --- | --- | --- | --- | --- | --- | --- | --- | --- | --- | --- | --- | --- |
|  |  |  | |  | |  |  | |  |  |  |  |  |  |  |  |  |  |  |  |  |  |
| Household & Woman ID | | | | | | | | | | |  |  |  |  |  |  |  |  |  |  |  |  |
|  | | |  | |  | | |  | | | Compound Number | | | | | | | | Household & Woman ID | | | |
|  | | | | | | | | | | |  |  |  |  |  |  |  |  |  |  |  |  |
