# Supplementary material for: Scaling-up the use of sulfadoxine-pyrimethamine for the preventive treatment of malaria in pregnancy: results and lessons on scalability, costs and programme impact from three local government areas in Sokoto State, Nigeria
Source: Malar J. 2016 Nov 4;15:533. doi: 10.1186/s12936-016-1578-x (PMC5097385; doi:10.1186/s12936-016-1578-x)
Supplement: Supplementary file 4 — Additional file 4. Unadjusted mean Head Circumference (HC) in millimeters (mm) among live newborns born between April and November 2015 (without October), and differences in mean HC by available variables. [file 12936_2016_1578_MOESM4_ESM.docx]

| **Supplemental File 4: Unadjusted mean Head Circumference (HC) in millimeters (mm) among live newborns born between April and November 2015 (without October), and differences in mean HC by available variables** | | | | |
| --- | --- | --- | --- | --- |
|  | n | **Mean HC in mm** | **SD** | **Pr > \|t\|** |
| **Sex of Infant** |  |  |  |  |
| Female | 2768 | 353.70 | 22.65 | <.0001 |
| Male (ref) | 2985 | 356.30 | 22.80 |  |
| **Intervention** |  |  |  |  |
| Yes | 3940 | 356.60 | 24.15 | <.0001 |
| No (ref) | 1821 | 351.70 | 19.07 |  |
| **SP dosage** |  |  |  |  |
| 0 | 2105 | 353.50 | 23.63 | <.0001 |
| 1 | 741 | 354.60 | 23.53 | 0.00 |
| 2 | 1445 | 354.95 | 21.60 | 0.00 |
| 3+ (ref) | 1470 | 357.51 | 22.09 |  |
| **Primigravida** |  |  |  |  |
| Yes | 1071 | 354.2 | 23.077 | 0.1649 |
| No (ref) | 4729 | 355.3 | 22.7762 |  |
| **At least 1 ANC visit** |  |  |  |  |
| Yes | 3215 | 355.60 | 20.63 | 0.03 |
| No (ref) | 2546 | 354.30 | 25.21 |  |
| **Month of Birth** |  |  |  |  |
| April | 755 | 360.50 | 22.89 | <.0001 |
| May | 887 | 355.49 | 20.92 | 0.54 |
| June | 838 | 353.06 | 20.86 | 0.11 |
| July | 766 | 354.53 | 23.78 | 0.79 |
| August | 809 | 354.31 | 22.61 | 0.65 |
| September | 860 | 352.98 | 26.17 | 0.09 |
| November (ref) | 846 | 354.83 | 21.14 |  |
| **Gestational Age at delivery** |  |  |  |  |
| 8 months | 17 | 340.88 | 26.32 | 0.00 |
| 9 months | 5656 | 354.99 | 22.84 | 0.04 |
| 10 months (ref) | 87 | 360.01 | 15.56 |  |
