# Supplementary material for: Scaling-up the use of sulfadoxine-pyrimethamine for the preventive treatment of malaria in pregnancy: results and lessons on scalability, costs and programme impact from three local government areas in Sokoto State, Nigeria
Source: Malar J. 2016 Nov 4;15:533. doi: 10.1186/s12936-016-1578-x (PMC5097385; doi:10.1186/s12936-016-1578-x)
Supplement: Supplementary file 5 — Additional file 5. Adjusted Associations between selected variables and the mean head circumference (HC) of newborns, between April and November 2015 (without October). [file 12936_2016_1578_MOESM5_ESM.docx]

| **Supplemental file 5: Adjusted Associations between selected variables and the mean head circumference (HC) of newborns, between April and November 2015 (without October)*** | | | |
| --- | --- | --- | --- |
|  | **Estimate** | **Standard Error** | **Pr > \|t\|** |
| **Intercept** | 364.62 | 3.09 | <.0001 |
| **Sex of Infant** |  |  |  |
| Female | -2.59 | 0.60 | <.0001 |
| Male (ref) |  |  |  |
| **Intervention** |  |  |  |
| Yes | 4.03 | 0.71 | <.0001 |
| No (ref) |  |  |  |
| **SP dosage** |  |  |  |
| 0 | -3.85 | 0.93 | <.0001 |
| 1 | -3.41 | 1.04 | 0.00 |
| 2 | -3.12 | 0.88 | 0.00 |
| 3+ (ref) |  |  |  |
| **Primigravida** |  |  |  |
| Yes | -1.23 | 0.77 | 0.11 |
| No (ref) |  |  |  |
| **At least 1 ANC visit** |  |  |  |
| Yes | 1.30 | 0.67 | 0.05 |
| No (ref) |  |  |  |
| **Month of Birth** | -0.83 | 0.15 | <.0001 |
| **Gestational Age at delivery** | |  |  |
| 8 months | -18.75 | 5.98 | 0.00 |
| 9 months | -2.96 | 2.45 | 0.23 |
| 10 months (ref) |  |  |  |
| * n=5,743; F-test p< 0.0001; Adj. r-sqare 0.023; RMSE: 22.52 | | | |
